# Computational Organic Chemistry: The Frontier for Understanding and Designing Bioorthogonal Cycloadditions

**DOI:** 10.1007/s41061-024-00461-0

**Published:** 2024-05-10

**Authors:** Dennis Svatunek

**Affiliations:** Institute of Applied Synthetic Chemistry, Technische Universität Wien (TU Wien), Getreidemarkt 9, 1060 Vienna, Austria

**Keywords:** Computational chemistry, Organic chemistry, Click chemistry, Density functional theory, Bioorthogonal

## Abstract

Computational organic chemistry has become a valuable tool in the field of bioorthogonal chemistry, offering insights and aiding in the progression of this branch of chemistry. In this review, I present an overview of computational work in this field, including an exploration of both the primary computational analysis methods used and their application in the main areas of bioorthogonal chemistry: (3 + 2) and [4 + 2] cycloadditions. In the context of (3 + 2) cycloadditions, detailed studies of electronic effects have informed the evolution of cycloalkyne/1,3-dipole cycloadditions. Through computational techniques, researchers have found ways to adjust the electronic structure via hyperconjugation to enhance reactions without compromising stability. For [4 + 2] cycloadditions, methods such as distortion/interaction analysis and energy decomposition analysis have been beneficial, leading to the development of bioorthogonal reactants with improved reactivity and the creation of orthogonal reaction pairs. To conclude, I touch upon the emerging fields of cheminformatics and machine learning, which promise to play a role in future reaction discovery and optimization.

## Introduction

In recent decades, computational chemistry has emerged as an indispensable tool in the advancement of chemical sciences [1], with capabilities extending from elucidating and predicting reactivity and selectivity to optimizing and engineering superior systems. The unique advantage of computational chemistry lies in its ability to investigate features of chemical systems that are challenging or impossible to observe experimentally, thereby making it more than a mere substitute for laboratory work. In essence, computational chemistry significantly enriches our understanding and accelerates innovation.

Bioorthogonal chemistry has not been immune to the influence of computational chemistry as the most prominent bioorthogonal reactions are all based on cycloadditions. These reactions are particularly well-suited for computational study for several reasons. One key factor is that they typically feature well-defined, straightforward single-step or occasionally two-step mechanisms. Another reason is that the simultaneous formation of two bonds in concerted cycloadditions effectively “locks in” the reactant conformations, simplifying (though by no means trivializing) the computational analysis. This characteristic has historically placed cycloadditions and other similar pericyclic reactions at the forefront of many computational chemistry investigations [2]. When synergistically combined with experimental approaches, the adoption of computational methods has proved highly impactful, providing substantial insights in the exploration and development of novel bioorthogonal tools.

This review is structured around computational models and methods that have found successful application in the field of bioorthogonal chemistry. I will first provide an overview of the most important computational chemistry analysis methods and concepts used in this field, before diving into different bioorthogonal chemistries and the computational studies that have been performed.

## Primer on Computational Techniques for Bioorthogonal Chemistry

In the following section, I outline some of the essential computational methods and analysis techniques commonly used in the field of bioorthogonal chemistry. These methods provide the backbone for interpreting and predicting reaction mechanisms, energetics, and selectivities that are central to bioorthogonal research. While this overview is concise, it aims to equip readers with a foundational understanding that will aid in understanding the discussions presented later in this paper.

### Computational Methods

A multitude of computational methods are available for use in bioorthogonal chemistry, which can be broadly categorized into wave function-based methods and density-based methods.

Wave function-based methods focus on approximating the Schrödinger equation to determine the system's energy and properties [3]. The Hartree–Fock (HF) method serves as the foundational technique in this category. However, Hartree–Fock is often deemed insufficiently accurate in modern computational chemistry primarily because it neglects electron correlation. While advanced methodologies, termed post-Hartree–Fock methods, aim to rectify this issue, each comes with its own set of strengths and weaknesses. For example, Møller–Plesset perturbation theory does account for electron correlation effects but may not capture them comprehensively. Therefore, other methods, such as coupled cluster techniques or configuration interaction, exist to address different aspects of electron correlation and other limitations, offering a range of options for various types of systems and problems. Despite the accuracy of these methods, their computational cost escalates rapidly, making them impractical for large, realistic systems.

In comparison, density functional theory (DFT) methods provide a more computationally efficient alternative [4, 5]. Grounded in the Hohenberg–Kohn theorem, DFT replaces the many-body wave function problem with a single-body problem related to electron density, thereby significantly reducing computational cost. The electron density serves as the fundamental variable to describe the system, while the density functional is employed to calculate energies and various properties from this electron density. DFT is highly effective for modeling cycloaddition reactions commonly encountered in bioorthogonal chemistry. In fact, DFT is almost exclusively employed in computational investigations of click reactions due to its accuracy and computational efficiency. However, DFT is not without its own drawbacks. Specifically, there is no hierarchical ranking to indicate which functional is universally better than another. The effectiveness of a given functional can vary dramatically depending on the particular system under investigation. This makes it difficult to predict a priori which functional will deliver the most accurate results for a specific problem. Consequently, benchmarking is essential, and researchers face the challenging task of selecting from a bewildering array of functionals, each with their unique strengths and limitations. These functionals go by designations such as PBE, M06-2X, ωB97X-D3, or B2PLYP, contributing to what is often called the alphabet soup of density functionals.

In addition to computational methods, it is also essential to have a representation for either the wave function or electron density. Basis sets comprise mathematical functions designed to approximate atomic wave functions in molecular systems. Gaussian-type functions are commonly used due to their computational efficiency, although Slater-type orbitals provide a more accurate approximation, but at a higher computational cost. Larger basis sets, which include more functions, generally offer greater accuracy by providing additional flexibility in describing electron orbitals. However, this increased accuracy comes at a computational cost. Much like density functionals, the performance of a basis set can vary depending on the system and the method employed.

In naming conventions, the computational method is listed first, followed by a slash, and then by the basis set. For example, M06-2X/def2-SVP uses the M06-2X functional and the def2-SVP basis set, while CCSD(T)/cc-pVTZ combines the CCSD(T) method with the cc-pVTZ basis set.

Solvent effects are generally not included in the computational methods described above, which typically consider molecules in isolation, often referred to as “gas-phase calculations”. To capture the influence of a surrounding medium such as a solvent, implicit solvent models are often employed. These models approximate the effects of solvation by surrounding the molecule with a continuous dielectric medium. Among the various implicit models, polarizable continuum model (PCM) [6, 7] and solvation model based on density (SMD) [8] are particularly well-regarded in the field. In terms of nomenclature, the solvent model used is often appended to the method description, such as PCM(methanol), to indicate that the PCM was employed with methanol as the solvent. However, it is worth noting that no universally accepted naming convention exists for denoting implicit solvent models.

It is important to note that density functional theory methods usually do not account for dispersion interactions, which are crucial in many chemical systems. Various approaches exist to include these effects, with Grimme's empirical dispersion corrections being among the most popular. These corrections, available in versions like D3 (third generation) [9] and the more recent D4 [10], are computationally inexpensive and highly effective. In computational nomenclature, the dispersion correction is often appended to the functional name. For example, B3LYP-D3(BJ) indicates the use of the B3LYP functional with Grimme's D3 dispersion correction and Becke-Johnson damping.

### Exploration of the Potential Energy Surface

A deep understanding of the potential energy surface (PES) is a cornerstone for any investigation of reaction mechanisms and reaction rates in organic chemistry. By exploring critical points on the PES, such as reactants, transition states, intermediates, and products, we gain invaluable insights into the thermodynamics and kinetics of chemical reactions. However, the value of the PES analysis is not confined to its stand-alone merits. Knowledge of the energy surface provides the basis for all other methods used in computational chemistry.

**Fig. 1:**
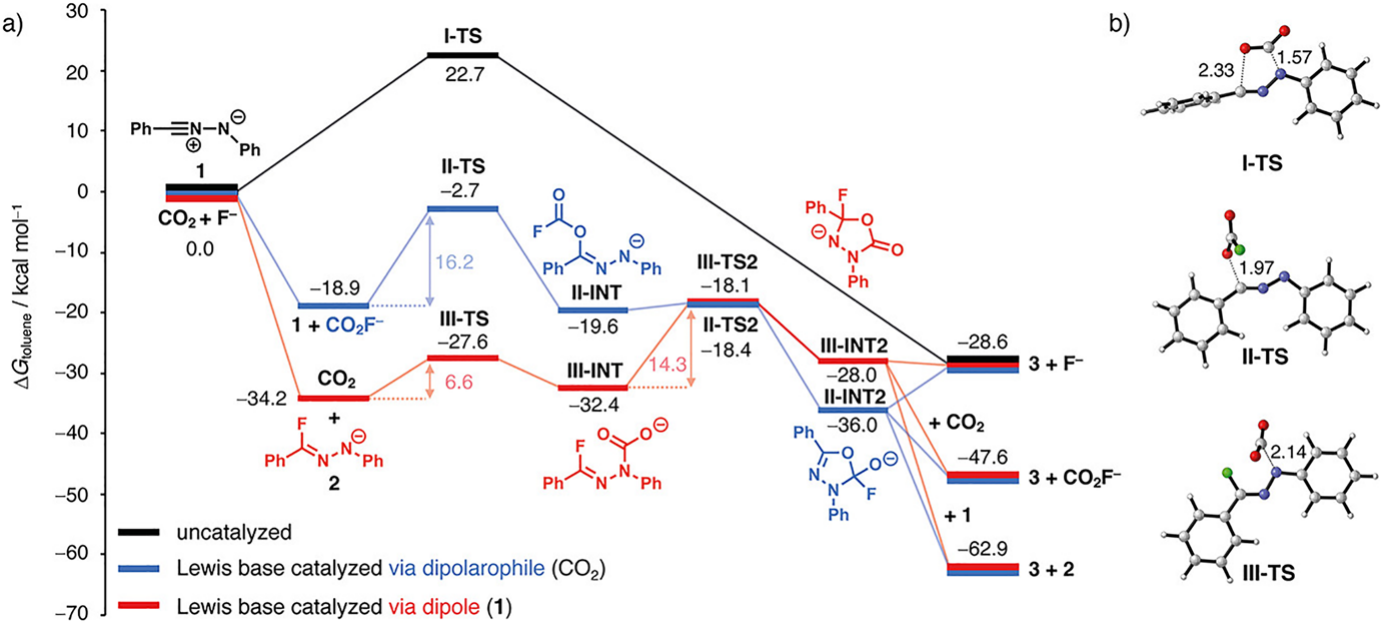
Example of a reaction profile in the study of a 1,3-dipolar cycloaddition. Three different pathways are considered. Reprinted from [11] with permission

Typically, the study of bioorthogonal cycloadditions involves the computation of geometries and energies of the starting materials, intermediates, products, and transition states to construct a comprehensive reaction profile (Fig. 1). This profile serves as the basis for applying transition state theory, whereby the height of the energy barriers can be translated into reaction rates. These rates provide insights into the reactivity of the system and, when multiple reaction pathways are evaluated, information about selectivity can also be inferred.

It is worth noting that the computational methods commonly employed, predominantly based on density functional theory, do not always reproduce absolute barrier heights accurately, often due to limitations in accounting for solvent effects. However, these methods generally perform well in reproducing relative barrier heights, thus enabling the calculation of relative rates.

The computation of reaction profiles is commonly used not only to predict reactivities but also to elucidate mechanisms by comparing various pathways. An inspection of the molecular geometries involved can already provide a preliminary understanding of the system's reactivity. However, to gain a deeper and more comprehensive understanding of the system, it is typically necessary to employ more detailed investigations using the methods described in the following sections.

### Orbital-Based Analysis

Molecular orbitals, while being theoretical constructs that are not observable, offer invaluable insights into the reactivity and selectivity of chemical reactions. One of the most well-known theories utilizing molecular orbitals is the frontier molecular orbital (FMO) theory, introduced by Kenichi Fukui [12], who received a Nobel Prize for this work. According to this theory, a reasonable prediction of the reactivity and selectivity of organic reactions can be made by focusing on the energies and shapes of the highest occupied molecular orbital (HOMO) and the lowest unoccupied molecular orbital (LUMO). In the context of cycloadditions, a small HOMO–LUMO gap is generally indicative of a reaction that will proceed rapidly [13]. However, it is important to note that there are exceptions, and in this review, I will discuss several bioorthogonal reactions that do not follow predictions from FMO theory.

Canonical molecular orbitals do not always align well with the concept of bonding in organic chemistry, which traditionally employs localized electron pair models, such as Lewis structures. However, canonical orbitals can be localized through unitary transformation, effectively creating orbitals that better resemble the bonding scenarios depicted in Lewis structures. Natural bond orbitals (NBOs) [14] are the most widely used type for this purpose, but other types, such as intrinsic bond orbitals (IBOs) [15] are also gaining traction. These localized orbitals can be used to identify bonding characteristics, and they also enable the analysis of charge transfer between bonding and antibonding orbitals.

In particular, the application of NBOs in investigating stereoelectronic effects, such as hyperconjugation, has yielded valuable insights into the reactivity of a wide array of organic reactions. This analysis method is extensively utilized in the field of bioorthogonal chemistry and has led to the development of quantitative models that facilitate the prediction of reactivity. Harris and Alabugin published an outstanding review on the analysis of stereoelectronic effects in bioorthogonal 1,3-dipolar cycloadditions [16].

### Distortion/Interaction Analysis and Energy Decomposition Analysis

One of the challenges in computational chemistry is translating complex wave-function or electron density-based results into easy-to-understand models that align with traditional organic chemistry frameworks. Two methodologies, distortion/interaction analysis [17, 18] (DIA, also known as the activation strain model) [19] and energy decomposition analysis (EDA) [20], effectively address this issue.

**Fig. 2:**
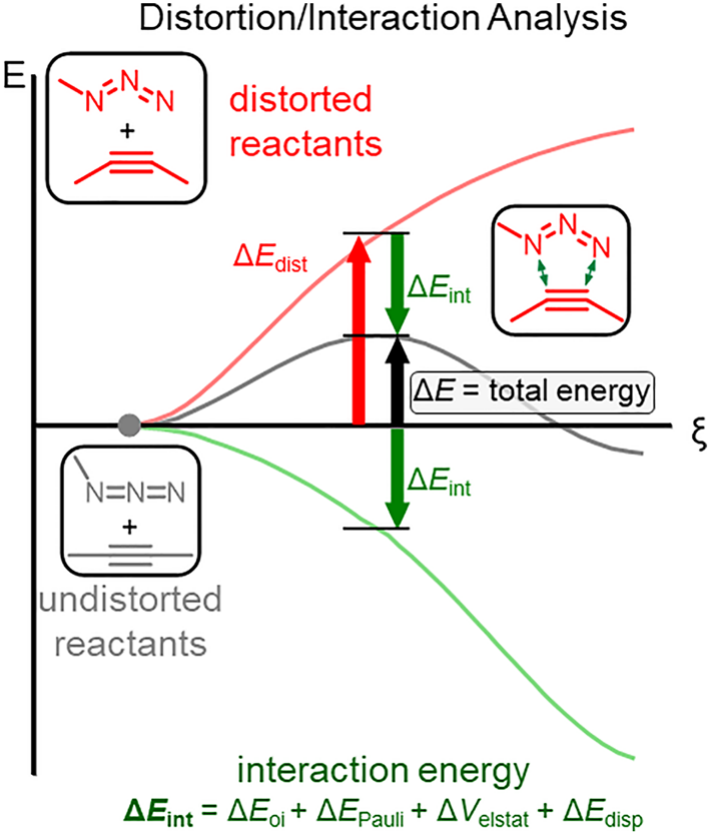
Schematic representation of the distortion/interaction analysis and energy decomposition analysis. Adapted from [21]

In DIA, when two “fragments” (which could be separate molecular species or segments of the same molecule) interact, the energy of this interaction is divided into two main components: the distortion energy (Δ*E*_dist_) and the interaction energy (Δ*E*_int_). The distortion energy quantifies the energy required to deform the fragments from their relaxed geometries into the geometries they adopt during interaction. The interaction energy, in contrast, represents the energy released when these deformed fragments come into contact (Fig. 2).

The interaction energy can be further broken down into chemically meaningful terms through various energy decomposition methods [20]. Multiple versions of this approach exist, each grounded in different foundational principles; examples include absolutely localized molecular orbitals EDA (ALMO-EDA), [22, 23] symmetry-adapted perturbation theory (SAPT) [24, 25], and the original EDA by Morokuma [26]. Although these methods yield similar yet distinct energy components, I will focus here on the EDA implementation in the ADF software package [27], as it is widely used in bioorthogonal chemistry. This particular method categorizes the energy into the following components: Pauli repulsion (Δ*E*_Pauli_), which represents the closed-shell repulsion between fragments; electrostatic interaction (Δ*V*_elec_), typically an attractive force; and orbital interaction (Δ*E*_oi_), which accounts for effects such as inter-fragment charge transfer and intra-fragment polarization.

DIA, both alone and in combination with EDA, has been shown to offer critical insights into cycloaddition reactions in general [11, 28–36]. Therefore, it is not surprising that these methods are extensively used in the field of bioorthogonal chemistry.

However, there are limitations to using DIA and EDA for studying cycloadditions. One significant challenge is choosing the appropriate structures for comparison. Traditionally, these analyses focus on the transition states of the reactions. Yet these cycloadditions often adhere to Hammond’s postulate, in which lower energy barriers correspond to earlier transition states. Earlier transition states, however, entail longer distances with less distortion and weaker interactions, including Pauli, electrostatic, and orbital interactions. As a result, comparing the energetics of transition states at varying positions along the reaction coordinate can yield skewed results [18, 37].

To address this issue, analyses are performed along a reaction coordinate where the same distance is compared across different systems [38–41]. An alternative strategy involves using “consistent geometries” with uniform bond lengths. While this approach has proven successful, it can be challenging when different levels of asynchronicity are observed, as it then becomes difficult to define a consistent geometry [42]. Consequently, the resulting numerical data must be interpreted with caution.

Recently, a novel analysis method that utilizes a two-dimensional (2D) surface of the potential energy surface has been proposed [43], although it has not yet been applied to cycloadditions.

### Other Analysis Methods

Although the computational techniques previously discussed are central to understanding bioorthogonal cycloadditions, there are other methods that, while less frequently employed, still offer valuable insights.

Conceptual density functional theory (CDFT) serves as a useful tool for quantifying reactivity descriptors such as chemical hardness and softness, and has been employed to understand the subtleties of reaction mechanisms [44]. Electron localization functions (ELF), on the other hand, provide a spatial representation of electron pairing within molecules, offering insights into the localization of electron density [45].

Noncovalent interaction (NCI) plots are another valuable resource, especially for visualizing and understanding weak intermolecular interactions, such as π–π stacking [46].

Additionally, charge and electrostatics analysis methods, such as Hirshfeld charge analysis [47] or electrostatic potential (ESP) plots [48], offer a different lens through which to view reactivity and molecular interactions. By evaluating the electric charge distribution within molecules, these methods shed light on the electrostatic interactions that may either facilitate or hinder reactions.

## (3 + 2) Cycloadditions

(3 + 2) cycloaddition reactions hold a unique position in bioorthogonal chemistry, offering diverse mechanisms and reactant pairs [49–52]. While the strain-promoted azide-alkyne cycloaddition (SPAAC) remains the most well-known example of these reactions, other variants also exist and contribute to the field. In this section I explore computational studies on various (3 + 2) cycloadditions, shedding light on their mechanisms, reactivity, and applicability in bioorthogonal contexts.

### Strain-Promoted Azide-Alkyne Cycloadditions

The strain-promoted azide-alkyne cycloaddition serves as a fundamental method in the field of (3 + 2) cycloadditions for bioorthogonal chemistry [50]. As the bioorthogonal equivalent to the copper-catalyzed azide-alkyne cycloaddition (CuAAC), often referred to as just “click reaction”, SPAAC offers a reasonably fast biocompatible reaction that has found a wide range of applications.

**Fig. 3:**
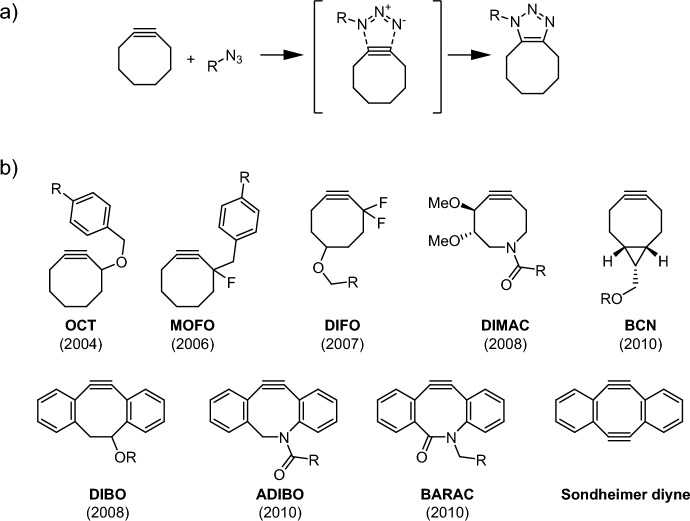
**a** Mechanism of the strain-promoted azide-alkyne cycloaddition (SPAAC). **b** Core structures of selected cyclooctyne derivatives developed for rapid bioorthogonal cycloadditions

In the SPAAC, an organic azide reacts with a strained alkyne, usually a cyclooctyne, in a (3 + 2) cycloaddition (Fig. 3a). Soon after its introduction as a bioorthogonal reaction in 2004 [53], optimization of the reaction began, and a range of different cyclooctynes were introduced (Fig. 3b) [54].

**Fig. 4:**
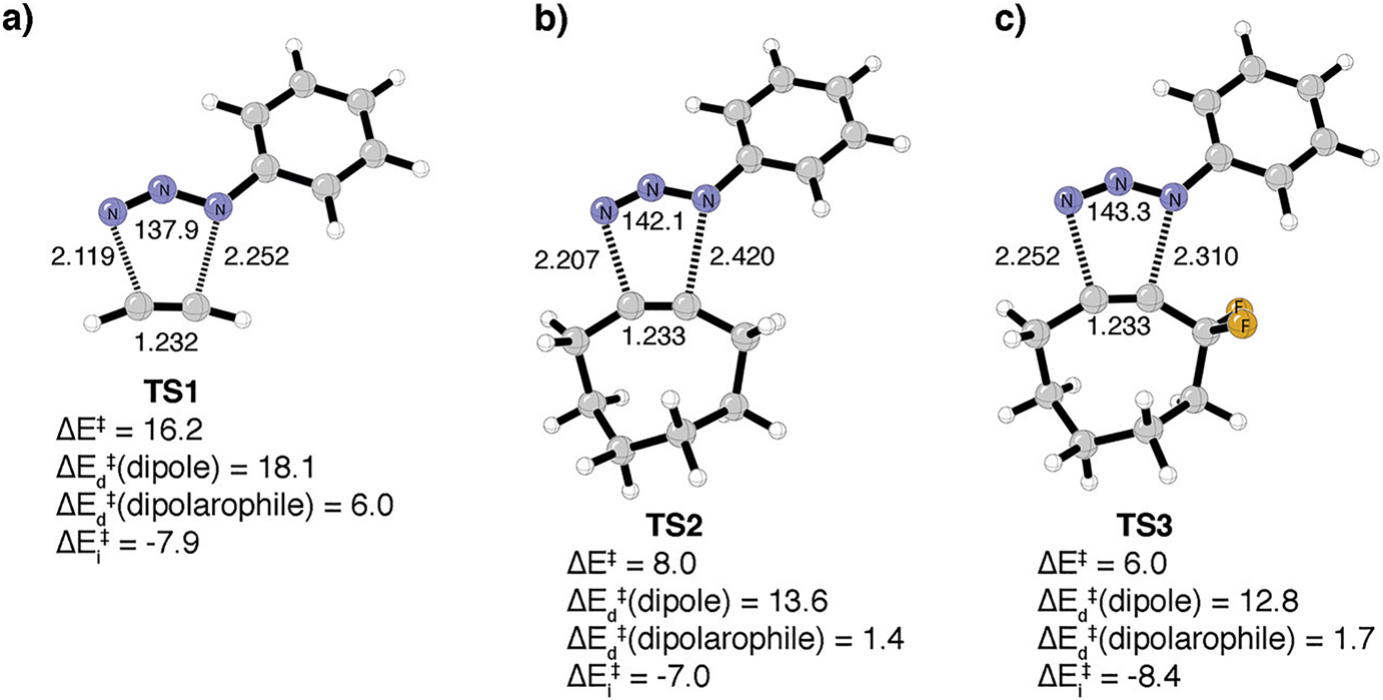
B3LYP/6-31G(d) Δ*E*^‡^, ΔE_d_^‡^(dipole), Δ*E*_d_^‡^(alkyne), and Δ*E*_i_^‡^ for the concerted transition structures of phenyl azide cycloaddition with **a** acetylene, **b** cyclooctyne, and **c** difluorocyclooctyne (kcal/mol). **TS3** is the lowest energy regioisomer. Reprinted with permission from [56]. Copyright 2008 American Chemical Society

In 2008, Houk and colleagues explored the high reactivity of cyclooctyne and the enhanced performance of the newly introduced "second-generation" cyclooctyne, **DIFO** [55], using distortion/interaction analysis (DIA) and frontier molecular orbital (FMO) theory [56]. Initially, they compared acetylene with cyclooctyne in their reactions with phenyl azide, as depicted in Fig. 4a, b. The DIA conducted at the respective transition states revealed that the elevated reactivity of the strained cyclooctyne primarily stems from a reduction in distortion energy. The authors acknowledged that this lower distortion energy is partially due to the earlier transition state but emphasized that the main contributing factor is the lowered distortion energy itself [55]. Today, performing an analysis along a reaction coordinate or using a consistent geometry would be the preferred methods (See section 2.4). However, such analyses were not commonly employed in 2008, which was shortly after the introduction of the DIA technique in 2007 [17]. For **DIFO**, the authors concluded that the electron-withdrawing substituents lead to enhanced orbital interactions, thereby resulting in a more favorable Δ*E*_int_, as illustrated in Fig. 4c.

In a follow-up study, the same research group looked deeper into the computational investigation of the reactivity of cyclooctynes like **DIFO** [57]. First, they examined the regioselectivity of cyclooctynes such as **DIFO** in reactions with phenyl azide. They found significant selectivity in gas-phase calculations; however, the inclusion of solvation effects negated this selectivity, in alignment with the experimental results. Next, the group showed that electron-withdrawing substituents in the 3-position of the cyclooctyne reduced the reaction barrier. They then compared the reactivity of cyclohexyne, cycloheptyne, cyclooctyne, and cyclononyne with phenyl azide and found that activation energies correlated well with distortion energies. However, it is important to note that this analysis was again carried out at the respective transition states. Furthermore, the series of cycloalkynes adhere to Hammond's postulate when reacting with phenyl azide; lower energy barriers correspond to earlier transition states, which could at least partially account for the observed correlation.

**Fig. 5:**
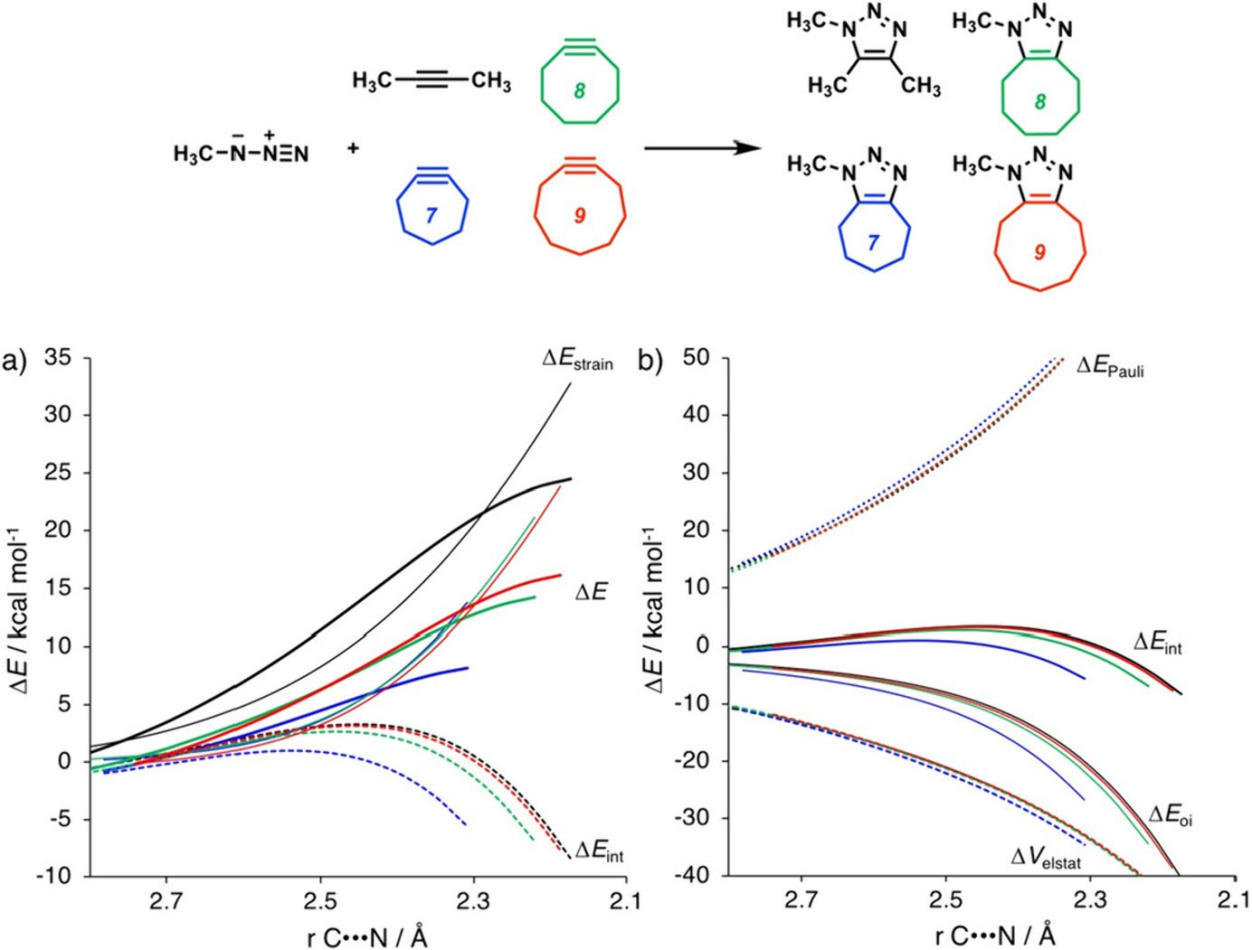
Distortion/interaction activation (DIA) strain analyses (**a**) and energy decomposition analyses (EDA) (**b**) of the cycloaddition reactions of Az with alkynes (black, **2yne**; blue, **7yne**; green, **8yne**; red, **9yne**). All data were computed at M06-2X/TZ2P//M06-2X/6–31 + G(d). Reprinted from [58]

In 2019, the research groups led by Houk and Bickelhaupt revisited this topic with a study focused on understanding how the cycloaddition reactivity of cycloalkynes is influenced by alkyne distortion [58]. In this work, the DIA and energy decomposition analysis (EDA) are performed along a reaction coordinate defined by one of the forming bond lengths. This approach eliminates the bias that arises from comparing earlier and later transition states. The study demonstrates that cycloalkynes are more reactive than linear alkynes, owing to both reduced distortion energy and increased interaction energy. Figure 5 displays the results of both the DIA and EDA for these reactions, plotted along a common reaction coordinate. In the DIA graph, the linear alkyne (represented by the black line) clearly exhibits significantly higher distortion energy, here termed Δ*E*_strain_. Cyclononyne has only a marginally increased interaction energy compared to the linear alkyne. However, the smaller cycloalkynes demonstrate substantially enhanced interaction energy, attributable to increased orbital interactions.

**Fig. 6:**
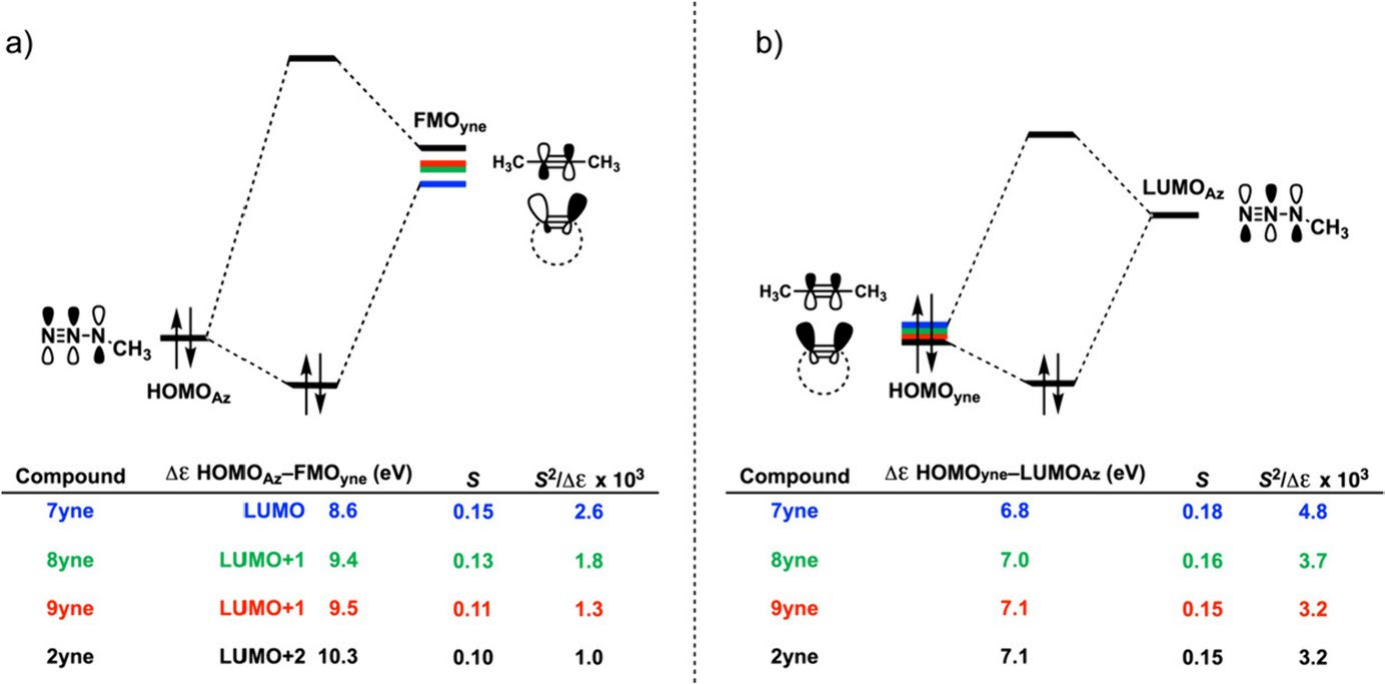
Molecular orbital (MO) diagram, with the key orbital energy gap, overlap, and the *S*^2^/Δ*ϵ* terms of **a** the HOMO_**Az**_–FMO_**yne**_ interaction and **b** the HOMO_yne_–LUMO_Az_ interaction for cycloaddition reactions between methylazide Az and alkynes **2yne** and **7yne**–**9yne**. All data were computed at M06-2X/TZ2P//M06-2X/6–31 + G(d).* FMO* Frontier molecular orbital,* HOMO* highest occupied molecular orbital,* LUMO* lowest unoccupied molecular orbital. Reprinted from [58]

The enhanced orbital interaction is attributed to a reduced FMO gap, primarily achieved by stabilizing the lowest unoccupied molecular orbital (LUMO) of the alkyne (Fig. 6). Additionally, increased orbital overlap is facilitated by geometric changes in both the highest occupied molecular orbital (HOMO) and LUMO of the cycloalkyne, which occur due to the bending of the triple bond. This mechanism is currently the accepted model for explaining the reactivity of cyclooctyne.

De Proft and coworkers arrived at a similar conclusion through the use of conceptual density functional theory (CDFT) [59]. These authors highlighted that the global softness, which can be thought of as a measure of a molecule's willingness or readiness to interact and react with other molecules, increases when the triple bond is bent more than 15°. Given that an increase in global softness correlates with a decreased HOMO–LUMO gap, these findings are consistent with those reported by Bickelhaupt and colleagues.

**Fig. 7:**
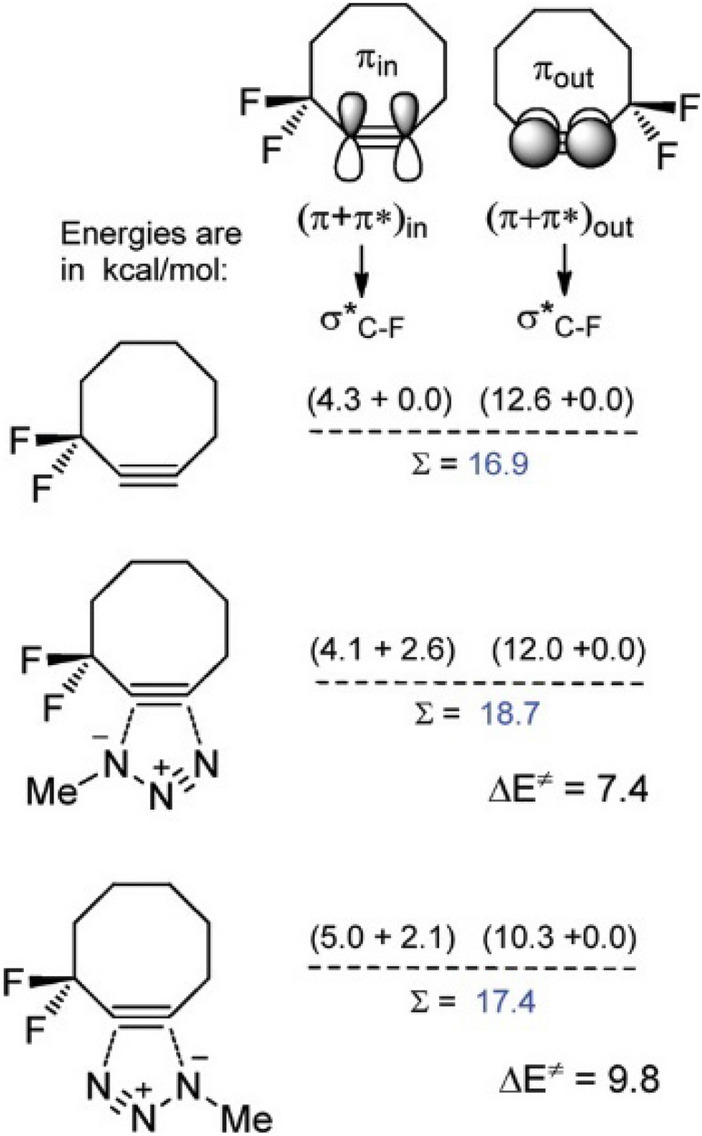
Hyperconjugative interactions between σ*C–F acceptor and the two π-systems in **DIFO** and two regioisomeric transition state structures (TSs) for its cycloaddition to methyl azide. The data are for natural bond orbital (NBO) second-order perturbation energies in kcal/mol at the B3LYP/6-31G(d) level. Δ*E*^⧧^ values at the same level of theory are given at the bottom of the figure. Reprinted with permission from [60]. Copyright 2011 American Chemical Society

Revisiting the subject of **DIFO**’s enhanced reactivity, Alabugin and colleagues investigated this topic in 2012, providing comprehensive insights into the mechanism at play [60]. They demonstrated that the propargyl fluorine atoms in **DIFO**, although not optimally aligned, can engage in hyperconjugation. Specifically, donation from the in-plane alkyne π-system to the σ*C–F orbital directly contributes to transition state stabilization. Figure 7 illustrates the energy stabilization via hyperconjugation between the π system of the alkyne and the antibonding σ* of the C–F bond. For the reactive π_in_ and the non-reactive π_out_, the σ*C–F is more optimally aligned with π_out_; however, its alignment remains largely unchanged during the reaction. Notably, π_in_ experiences enhanced hyperconjugation at the transition state, which particularly stabilizes the 1,5-addition by improving the ability of the alkyne to act as an acceptor. This hyperconjugation is also seen as making the alkyne more flexible and allowing for easier distortion, although this effect is smaller in magnitude. Additionally, the fluorine atoms serve as hydrogen bond acceptors and can interact favorably with CH groups.

**Fig. 8:**
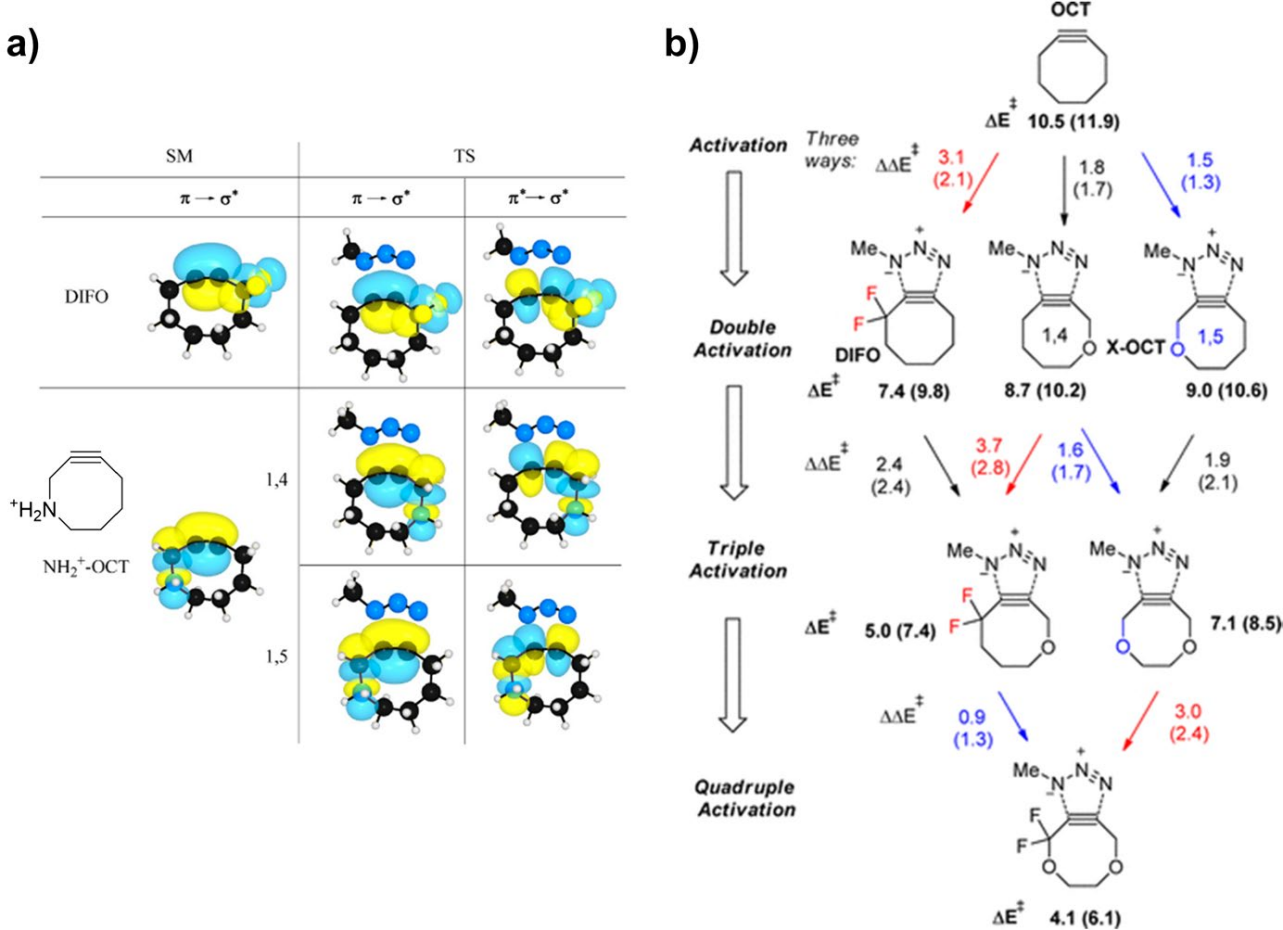
**a** NBO plots for orbital interactions between the propargylic σ-acceptors and the reacting in-plane alkyne π-bond in the cycloaddition TSs of difluorocyclooctyne and **NH**_**2**_^**+**^**-OCT** with methyl azide. **b** Modularity and cooperativity for the three transition state stabilization approaches for cycloadditions of substituted cyclooctynes and methyl azide. B3LYP/6-31G* activation energies (Δ*E*^⧧^) are given in bold, changes in the activation barriers (ΔΔ*E*^‡^) introduced by an additional level of activation are given near the arrows. Conductor-like polarizable continuum model (CPCM; water) solvation corrections are given in parentheses. Adapted with permission from [61]. Copyright 2012 American Chemical Society

However, the team noted that an optimal antiperiplanar alignment of the σ* of the C–F bond would increase reactivity even further. Building on this, Gold et al. designed new cyclooctynes using endocyclic hyperconjugation acceptors that align perfectly with the reactive π_in_ orbital [61]. They describe different factors that can enhance the reactivity of alkynes when reacting with azides. Primarily, incorporating the alkyne into an 8-membered ring introduces strain, accelerating the reaction by destabilizing the reactant. However, excessive strain can negatively impact the stability of the reactant. The presence of a geminal exocyclic difluoro, as in **DIFO**, offers subtle hyperconjugative support and electrostatic CH···F interactions. Additionally, the introduction of two endo-cyclic acceptor σ*C–X orbitals provides hyperconjugative assistance. Incorporating oxygen or NH_2_^+^ into the cyclooctyne decreases the ring strain, yet it still enhances reactivity. Figure 8a displays natural bond orbital (NBO) plots that depict hyperconjugative orbital interactions between propargylic σ-acceptors and the in-plane alkyne π-bond during the cycloaddition transition state structures (TSs) of difluorocyclooctyne and **NH**_**2**_^**+**^**-OCT** with methyl azide.

Including all four possible modes of action, quadruple activation can be achieved. Figure 8b shows the different modes of activation and the calculated reduction of activation energy.

**Fig. 9:**
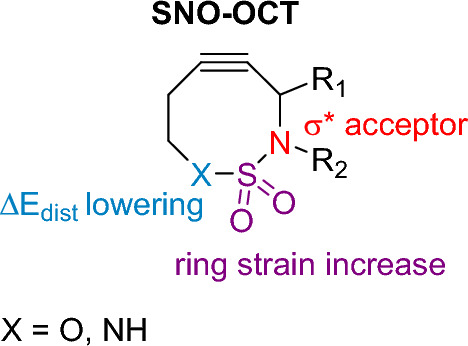
Structural depiction of **SNO-OCT**, highlighting elements integral to its reactivity

The Shomaker and Raines teams drew upon insights from previous studies by Raines and his colleagues, as well as research from Alabugin and his team, to develop a cycloalkyne that boasts enhanced reaction rates while maintaining stability [62]. This was achieved by implementing electronic activation using heteroatoms at both the propargylic and homopropargylic positions, culminating in the creation of **SNO-OCT**, as depicted in Fig. 9. This particular cyclooctyne has incorporated within its structure a sulfamate or sulfamide group. According to the authors, this group accelerates the cycloaddition reaction through several intertwined mechanisms. First, the propargylic nitrogen functions as a σ*C–N acceptor, which promotes hyperconjugation, and this effect is further intensified by the presence of the electron-withdrawing SO_2_ unit. Additionally, the X group, represented in Fig. 9 where X can be either O or NH, increases reactivity. This is attributed to the hybridization effect that reduces distortion energies during the reaction's progression. Lastly, by including the sulfamide or sulfamate group, there's a modulation in the ring strain, which subsequently results in a reduced Δ*E*_dist_.

**Fig. 10:**
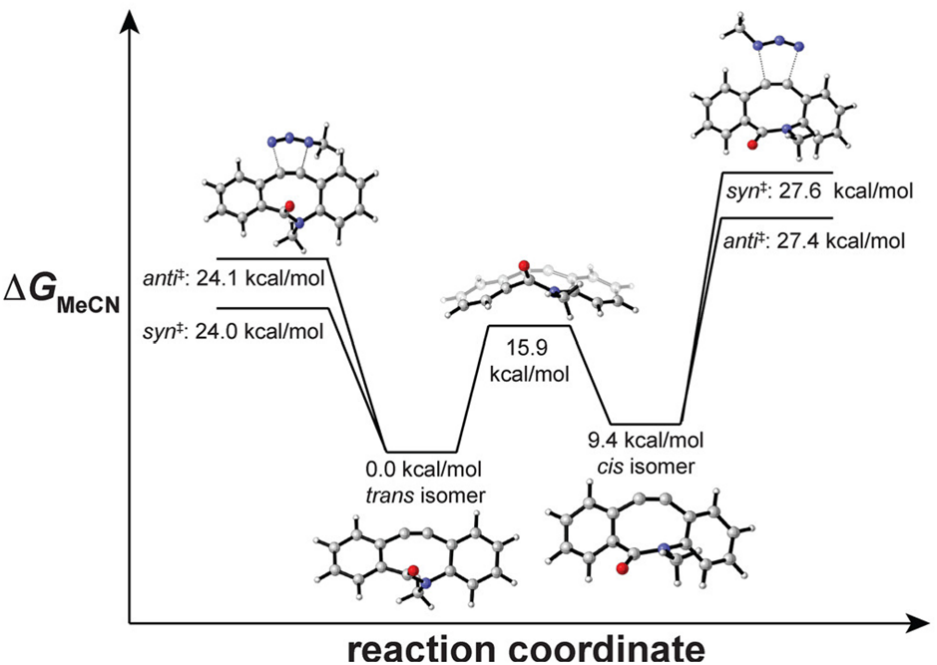
Under Curtin-Hammett conditions, **BARAC**’s amide conformation influences reactivity and regioselectivity. The reaction coordinate diagram displays calculated activation free energies for reaction of the parent **BARAC** compound with methyl azide in acetonitrile. Also shown are the relative energies of* cis*- and* trans*-**BARAC** and the barrier to* cis/trans* interconversion. Transition state images show only the lowest-energy regioisomers. Reprinted with permission from [63]

Contrary to the approach of electronic activation, several benzo-substituted cyclooctynes have been developed. The increased reactivity of these compounds is attributed to increased strain induced by the sp^2^ hybridized carbons. Many of these cyclooctynes find frequent use in experimental work, with a handful even being commercially available. In 2012, the Bertozzi and Houk teams explored the high reactivity of dibenzocyclooctynes, especially **BARAC** [63]. Their findings indicated that the process of benzoannulation results in stronger bending of the alkyne, a characteristic directly linked to increased reactivity (vide supra). Their research also dealt with the exceptionally high reactivity of **BARAC**, especially considering the role of the amide bond in its structure. It was identified that the amide bond exists in a* trans* configuration, which helps in reducing strain. In contrast, a* cis* configuration would have been associated with considerably enhanced reactivity, as depicted in Fig. 10. In a subsequent study, Hosseinzadeh and colleagues computationally explored a derivative of **BARAC**, reaffirming the significant impact of conformation [64]. While the potential exists to design more reactive dibenzocyclooctynes using this design principle, it is important to note that compounds like **BARAC** lack bench stability [65].

**Fig. 11:**
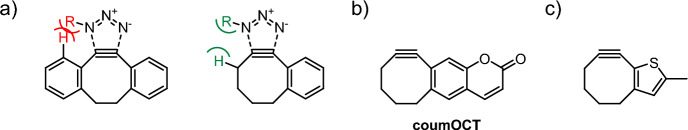
**a** Illustration of the steric clash in dibenzocyclooctyne derivatives as described by Goddard and coworkers [66], contrasting with the lack of such an interaction in mono benzoannulated derivatives. **b** coumOCT as detailed by Wong and coworkers[67]. **c** Thiophene-annulated version of cyclooctyne

In 2009, Goddard and his team introduced benzocyclooctynes as an alternative to dibenzo derivatives [66]. They believed that the flagpole hydrogens present in dibenzocyclooctynes could create steric hindrance with even small azides. However, incorporating just a single benzo group could achieve a similar rate acceleration, and the 1,4-addition would avoid the steric interference (as depicted in Fig. 11a). Building on this concept, Wong and colleagues presented a specific derivative, **coumOCT**, in 2017 (shown in Fig. 11b) [67]. They determined its rate constant with benzyl azide in CD_3_CN at 25 °C to be 0.012 M^−1^ s^−1^, a value aligning closely with the reactivity observed for **BCN** in other studies [21, 68].

**Fig. 12:**
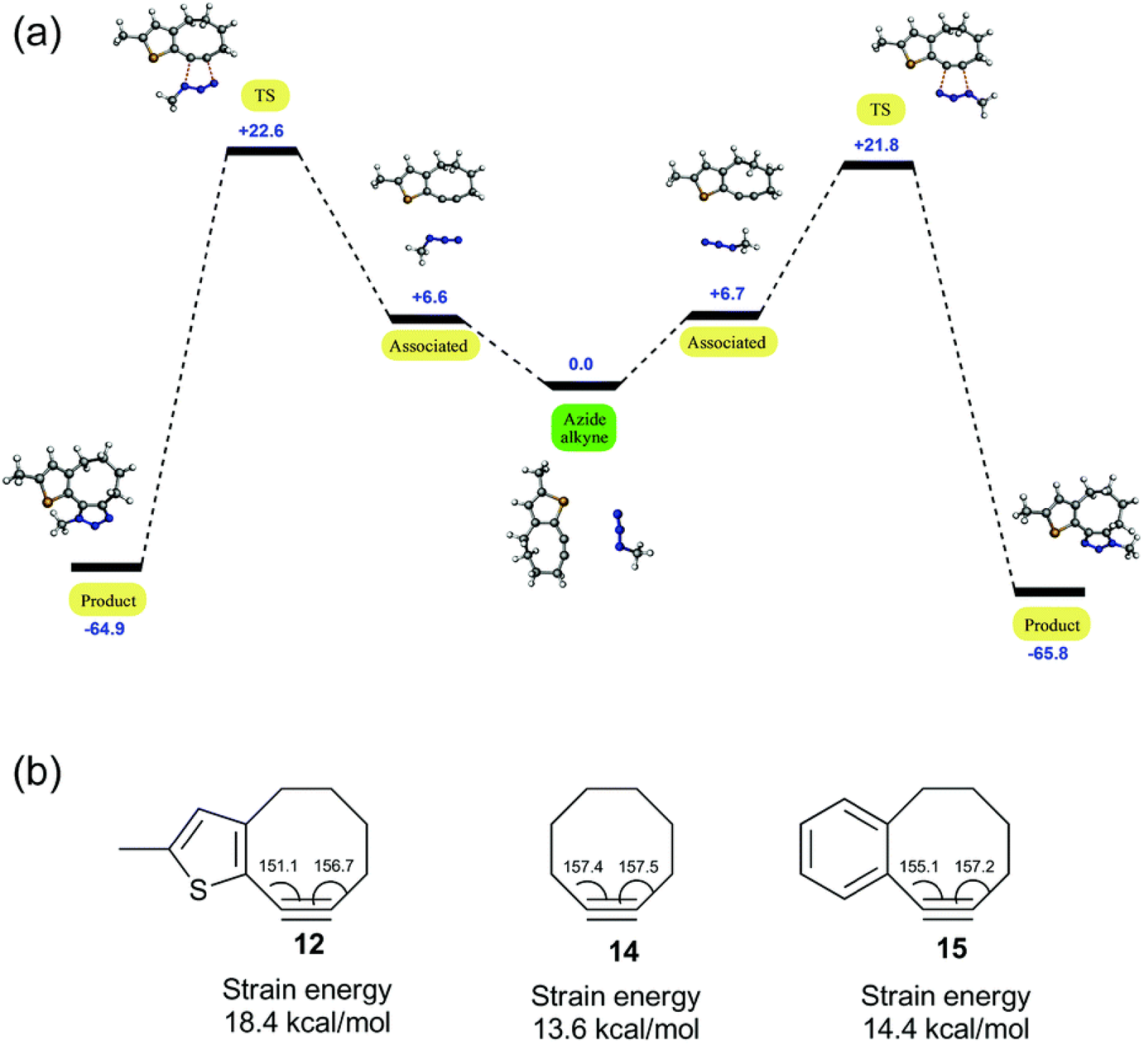
**a** Energy level diagram depicting strain-promoted azide-alkyne cycloaddition (SPAAC) between thiophene-annulated cyclooctyne **12** and methyl azide in methanol (energy in kcal mol^−1^). **b** Calculated strain energies and alkyne bond angles of 12, cyclooctyne 14, and benzo-fused cyclooctyne 15. Reprinted with permission from [69]

Spring and his team illustrated that it is not just benzo annulation that enhances reactivity; thiophene derivatives can also play a role [69]. Through both experimental and computational analyses, they found that cyclooctynes with thiophene derivatives (Fig. 11c) react rapidly with azides. Computational results reveal that the annulation with thiophene causes a stronger deviation from the linearity of the alkyne, as visualized in Fig. 12. This aligns with the understanding that a more bent alkyne has increased reactivity. Furthermore, they confirmed the hypothesis posited by Goddard and his team: mono-annulation results in reduced steric clash, making the 1,4-derivative the primary product.

**Fig. 13:**
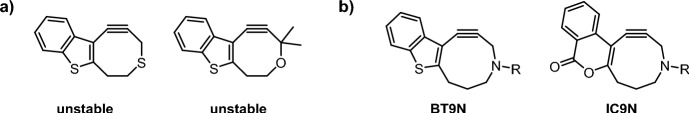
**a** Benzothiophene annulated cyclooctynes. **b** Cyclononynes introduced by Balova, Bräse, and colleagues

The two main strategies to enhance cyclooctyne reactivity involve either increasing strain through benzoannulation or utilizing hyperconjugative assistance to stabilize the transition state. In their quest for a reactive yet stable cycloalkyne, Balova, Bräse, and colleagues combined these approaches [70]. However, they discovered that cyclooctynes annulated with benzothiophene are unstable (Fig. 13a).

**Fig. 14:**
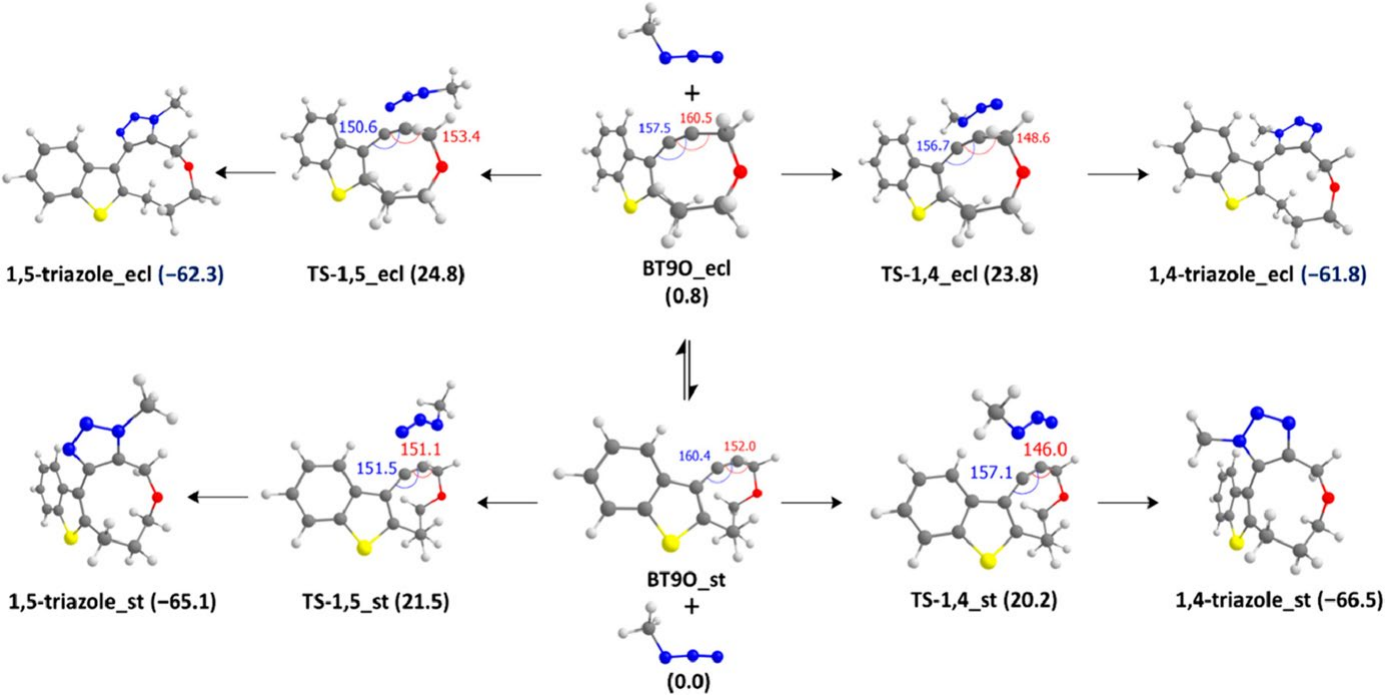
Calculated geometries and relative Gibbs free energies (kcal/mol, 298 K) for energy profile for cycloaddition of MeN_3_ and staggered and eclipsed conformations of **BT9O**. Reprinted with permission from [70]

However, a joint computational and experimental study revealed that 9-membered rings (Fig. 13b) display reactivity comparable to classical SPAAC reactions. The annulated ring is pivotal for the high reactivity of cyclononynes. This is attributed to the annulated ring's ability to maintain the alkyne bending in the staggered conformation of cyclononynes and provide additional activation. This activation arises from transition state stabilization due to stereoelectronic effects from an endocyclic heteroatom (π_in_* → σ*) and an adjacent fused ring (π_Het_* → π_in_*). Figure 14 illustrates the analysis of the eclipsed (ecl) and staggered (st) ring conformations of cyclononyne **BT9O**.

**Fig. 15:**
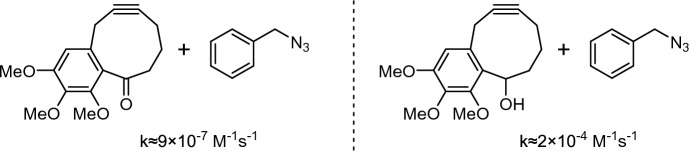
Reactivity of cycloalkynone and cacloalkynol investigated by Dudley and coworkers [71]

In 2012, Dudley and coworkers studied cycloalkynones as potential dipolarophiles [71]. Interestingly, the cycloalkynone examined exhibited reactivity that was two orders of magnitude lower than its corresponding alcohol (Fig. 15). This reduced reactivity was traced back to a transannular π → π* interaction between the alkyne π orbital and the carbonyl antibonding orbital, characterized by a Bürgi-Dunitz-like angle between the carbonyl group and the alkyne. This example underscores the importance of carefully considering even remote groups, as they can significantly affect the reactivity of cycloalkynes.

**Fig. 16:**
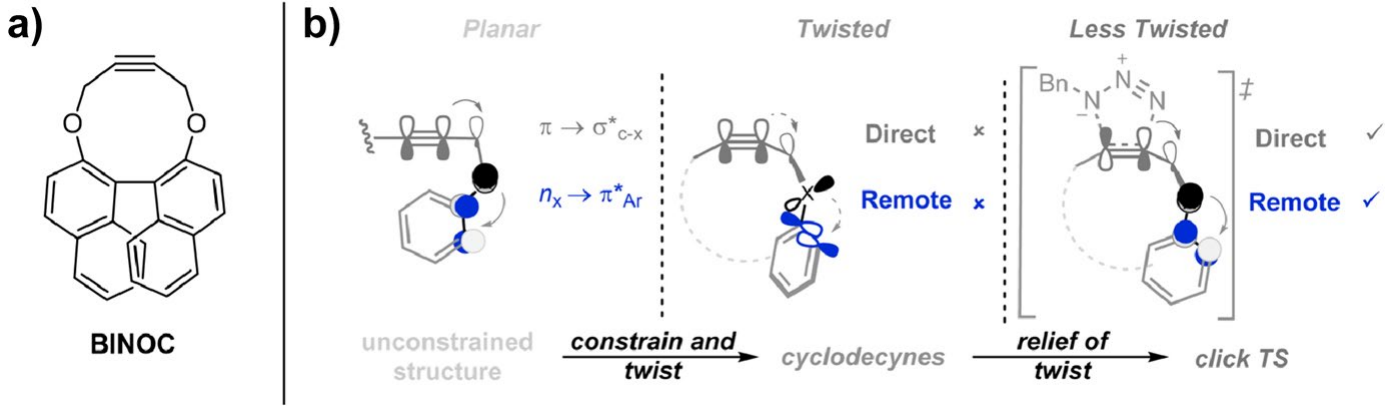
**a** Structure of **BINOC**. **b** Structural changes in the backbone of cyclodecynes turn on direct and remote electronic effects. Reprinted with permission from [72]

Alabugin and colleagues leveraged the influence of remote groups in designing cyclodecynes (Fig. 16) [72]. Cyclodecynes typically exhibit very low reactivity towards azides, making stereoelectronic activation crucial for enhancing their reactivity. These authors employed a dual strategy for this purpose. Similar to the previously mentioned cases with **NH**_**2**_^**+**^**-OCT** and **SNO-OCT**, endocyclic heteroatoms in propargylic position were used to directly activate the alkyne π system through π → σ*_C-X_ hyperconjugation. Additionally, a mechanism for remote activation was introduced by incorporating an aromatic system adjacent to the heteroatom, enabling n_X_ → π *_Ar_ stabilization. However, the presence of these interactions in both the starting material and the transition state would not affect the activation barrier. Here the authors very cleverly introduced constrained twists in the backbone so that neither stabilization is possible in the reactant. At the transition state, this twist is partially relieved, allowing for the necessary orbital alignment for targeted stabilization of the transition state, thus lowering the barrier height significantly in compounds such as **BINOC**.

**Fig. 17:**
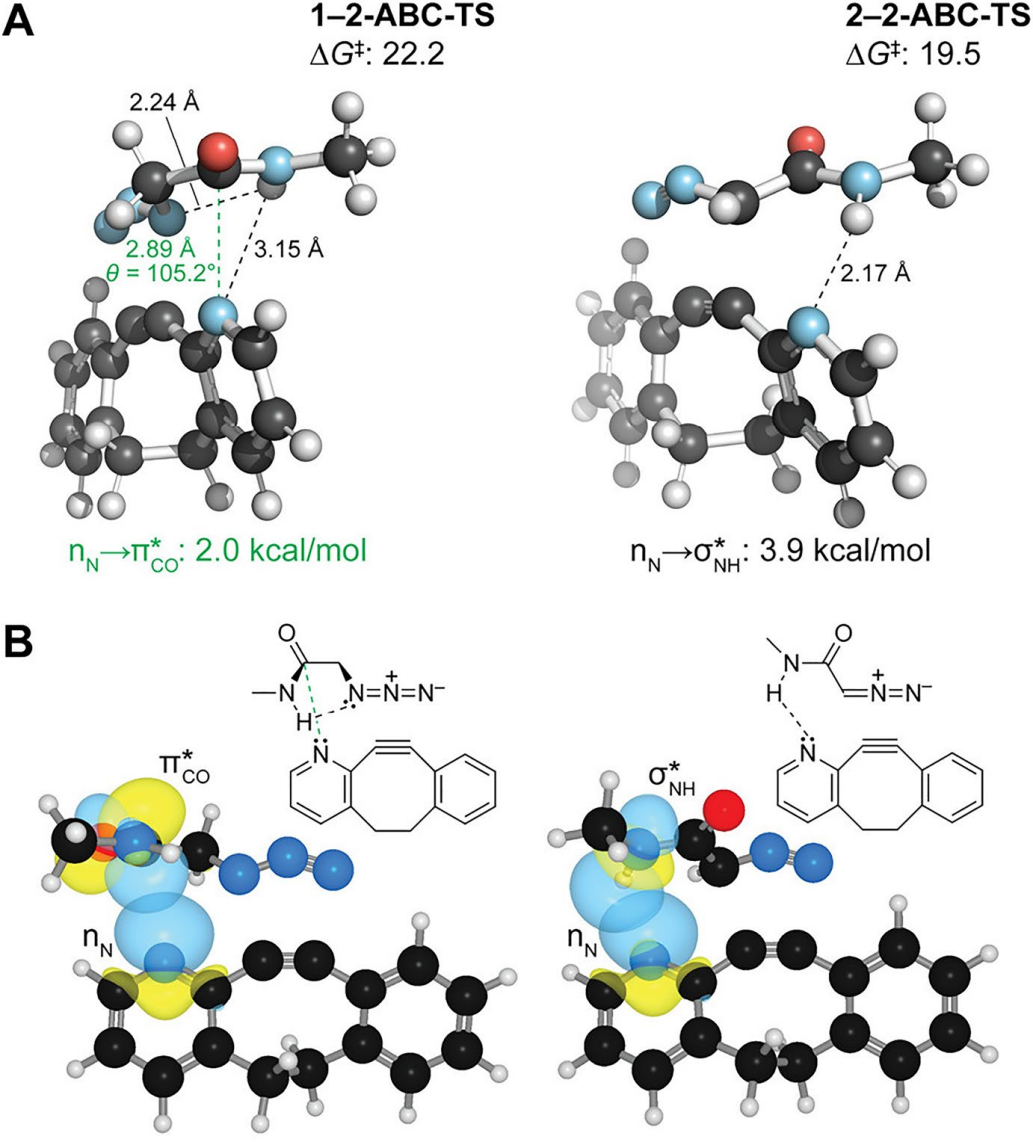
Comparison of interactions in **2-ABC** cycloadditions with *N*-methylazidoacetamide (**1**) and *N*-methyldiazoacetamide (**2**). **a** Second-order perturbations obtained from an NBO analysis. **b** Key stabilizing orbital interactions: N·C = O n → π* interaction with azide **1** and N·H–N hydrogen bond with diazo compound **2**. Reprinted with permission from [73]. Copyright 2021 American Chemical Society

Raines, Gold, and their teams presented **2-ABC**, a dibenzocyclooctyne that features a propargylic nitrogen atom (Fig. 17) [73]. This nitrogen atom facilitates dipole-specific n_N_ → π_CO_ or hydrogen bonding interactions with suitable azides or diazoacetamide dipoles. Such interactions boost the reaction rates, ranging from 30- to 1200-fold faster than **DIBO**. Moreover, these interactions result in high stereoselectivity during the cycloaddition. Figure 17 displays the transition states of **2-ABC** with an azide (on the left) and diazoacetamide (on the right). The NBO analysis highlights the n → π and hydrogen bond interactions.

**BCN**, introduced in 2010, is a highly reactive cyclooctyne [74]. Unlike others, its enhanced reactivity is not grounded in dibenzoannulation or electronic effects. Instead, a fused cyclopropane within the cyclooctyne backbone increases its reactivity. An in-depth study by the laboratories of van Delft and Bickelhaupt, using experimental and computational approaches looked into this cyclooctyne's reactivity [75]. Their findings revealed that the reaction proceeds with inverse electron demand, where the **BCN** HOMO interacts with the azide LUMO. Consequently, azides with electron-withdrawing substituents can accelerate the process, leading to rate constants that are up to 30-fold faster than conventional SPAAC.

**Fig. 18:**
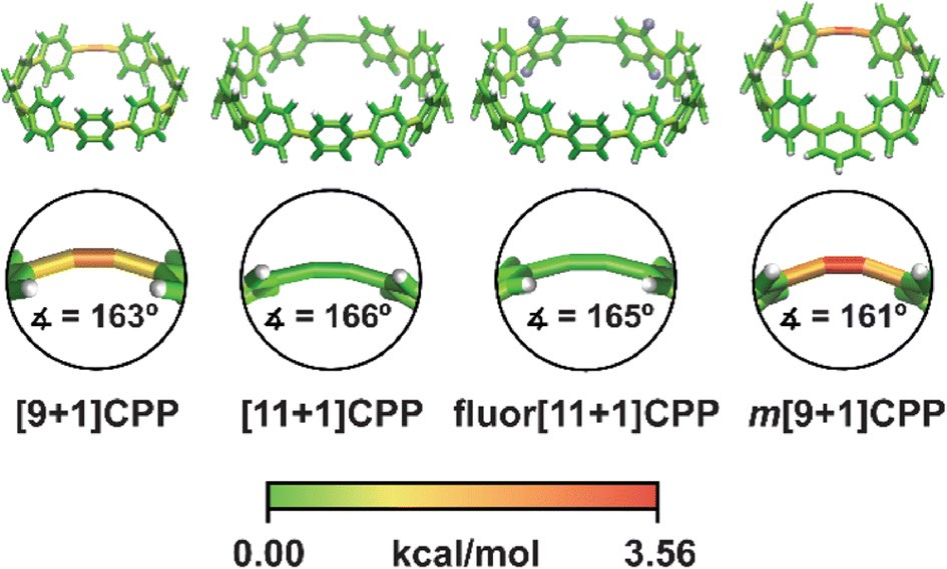
Strain analysis of the **[*n* + 1]CPPs** described in this study with the StrainViz computational tool.* CPPs* Cycloparaphenylenes. Reprinted with permission from [76]

Theory suggests that any strained triple bond should demonstrate good reactivity in cycloadditions with azides. In line with this, Jasti, Lopez, and their teams explored the reactivity of alkyne-containing cycloparaphenylenes (CPPs) [76]. Through variously sized cycloparaphenylenes incorporating an alkyne into their ring structure, they highlighted that a specific structure, m[9 + 1]CPP, with nine phenyl units and one meta substitution, exhibits high click reactivity. Their computational analysis employed the StrainViz method [77], allowing for the analysis of macrocyclic compounds and the visualization of local strain. This method showed that m[9 + 1]CPP experiences significant strain at the triple bond, characterized by a bond angle of 161° (Fig. 18).

**Fig. 19:**
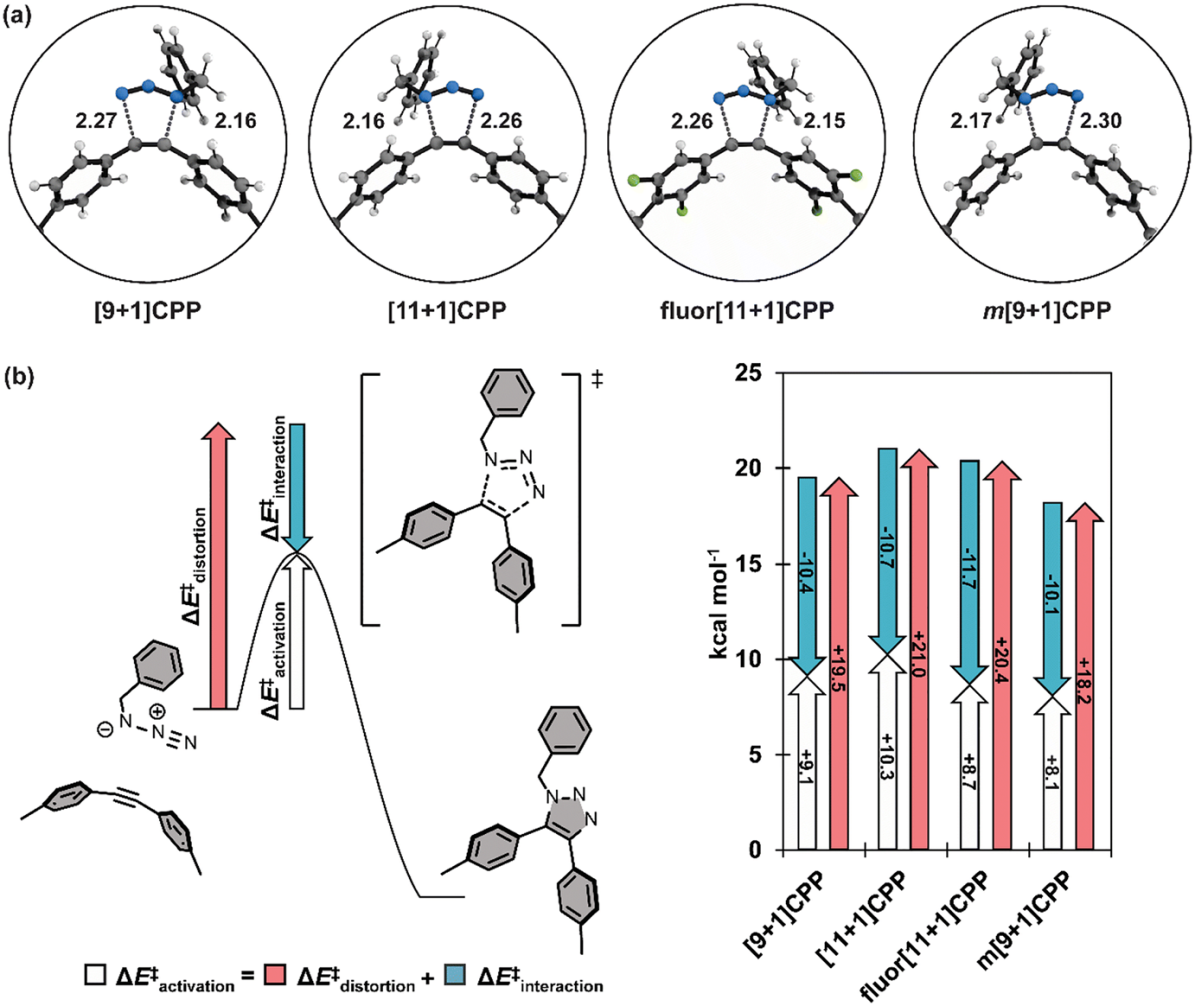
**a** Transition structures for each SPAAC reaction with benzyl azide; distances are shown in Angstroms (Å). **b** Generalization of the distortion–interaction model and Δ*E*^‡^_activation_ of each SPAAC reaction broken down into its Δ*E*^‡^_distortion_ and Δ*E*^‡^_interaction_ components. Reprinted with permission from [76]

The authors conducted a distortion/interaction analysis, as depicted in Fig. 19, which demonstrated that the added strain in m[9 + 1]CPP reduces the distortion energy.

**Fig. 20:**
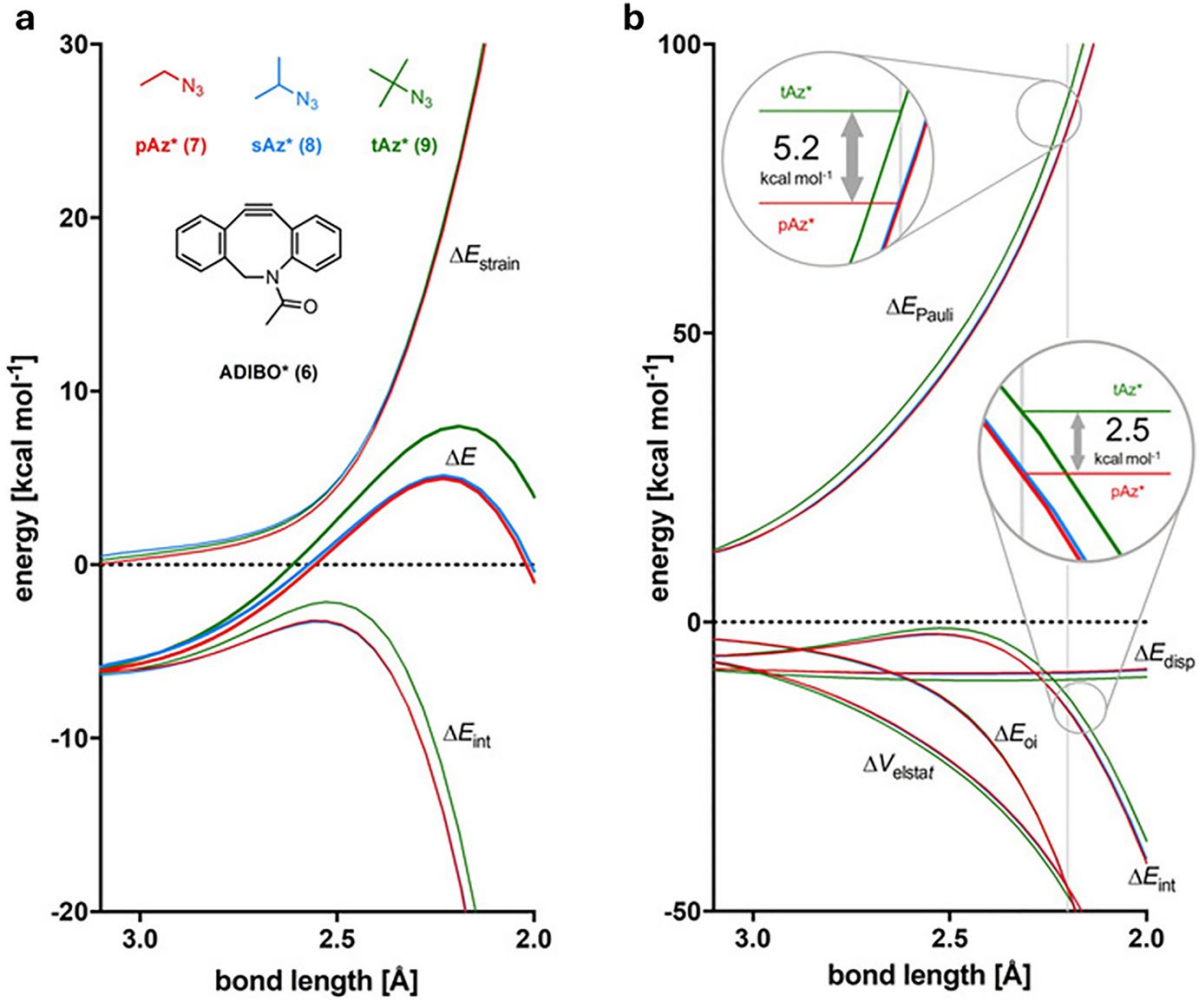
Activation strain analyses (**a**) and energy decomposition analyses (**b**) for the reactions **6** + **7** (red), **6** + **8** (blue), and **6** + **9** (green). Differences in Δ*E*_Pauli_ (5.2 kcal mol^−1^) and Δ*E*_int_ (2.5 kcal mol^−1^) between reactions **6** + **7** and **6** + **9** are indicated for a bond length of 2.2 Å (vertical gray line)

While the preceding discussion primarily centered on the cyclooctyne, the azide can also significantly impact reactivity. Denk and colleagues explored fluoroalkyl azides for potential application in in vivo click chemistry, specifically for radiolabeling. They found that primary, secondary, and even sterically demanding tertiary azides readily react with **BCN** [78]. Following this, Svatunek and colleagues demonstrated that when dibenzocyclooctynes, like **ADIBO**, are employed, there is a noticeable decline in reactivity with tertiary azides [21]. Computational analyses using DIA and EDA pinpointed the steric clashes between the tertiary azide and the **ADIBO** benzo groups as the cause for this reduced reactivity. Figure 20 displays both the DIA and EDA plotted against a reaction coordinate. The EDA indicates that, when compared at equivalent distances, the Pauli repulsion for the tertiary azide is strongly increased.

### Nitrone Cycloadditions

**Fig. 21:**
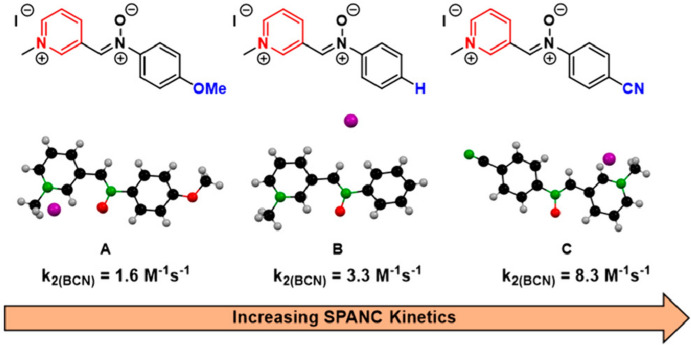
Nitrones investigated by Workentin and coworkers.* SPANC* Strain-promoted alkyne–nitrone cycloaddition. Reprinted with permission from [80]. Copyright 2019 American Chemical Society

In addition to azides, other 1,3-dipoles can participate in bioorthogonal (3 + 2) cycloadditions. One notable example are nitrones, which have been utilized in reactions with strained alkynes. This reaction is commonly referred to as the strain-promoted alkyne–nitrone cycloaddition (SPANC) [79]. Workentin and colleagues investigated the SPANC between electron-deficient pyridinium nitrones and **BCN** [80]. Their studies were inspired by findings from Dommerholt and coworkers who discovered that **BCN** reacts rapidly with electron-deficient azides [75]. Their research demonstrated that these electron-poor nitrones indeed react rapidly with **BCN**. A computational study highlighted HOMO_BCN_-LUMO_nitrone_ gaps of under 1 eV for these compounds, elucidating the observed high reactivity (Fig. 21).

Pezacki and team investigated the reactivity of nitrones further using computational techniques, specifically density functional theory (DFT) and DIA [81]. Their findings indicated that DIA serves as a valuable tool in understanding nitrone reactivity, showing that nitrone distortion energies correlate well with reaction barrier heights.

**Fig. 22:**
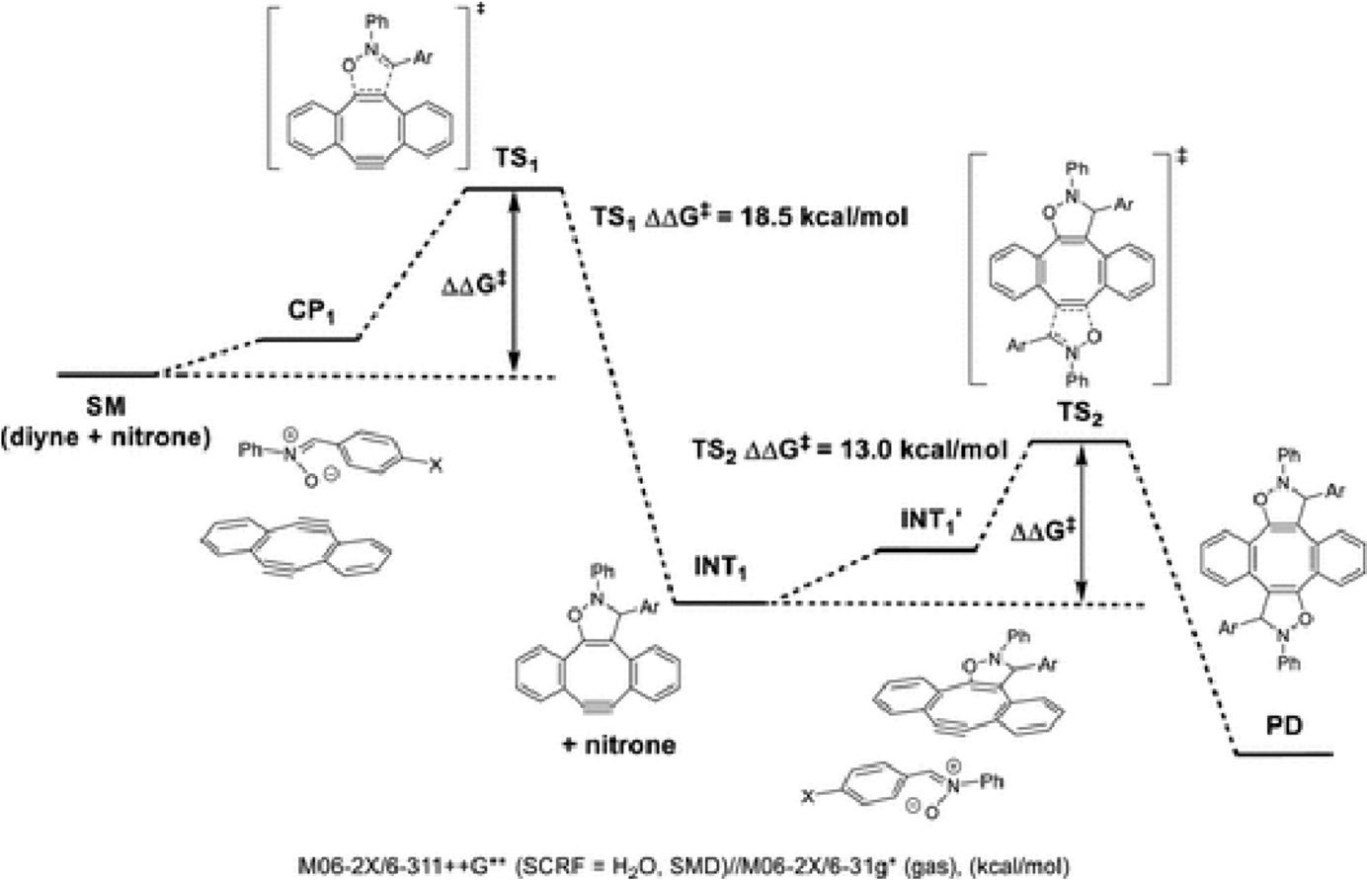
Calculated reaction coordinate diagram for the cycloaddition between nitrone dipoles and cyclooctadiyne dipolarophiles. The activation energies are shown for X ═ H. Reprinted with permission from [82]. Copyright 2023 American Chemical Society

In a more recent exploration, the same group investigated the double strain-promoted alkyne–nitrone cycloadditions utilizing the Sondheimer diyne [82]. This unique diyne has the capacity to react with two units of nitrone, essentially functioning as a crosslinking agent. Experimental kinetic analyses revealed that the subsequent reaction is faster than the initial one. DFT computations validate this finding, indicating a considerably reduced activation barrier for the second addition (Fig. 22). The authors propose that this increased reactivity results from the localization of the alkyne LUMO at the singular triple bond in intermediate **INT**_**1**_, as opposed to a more extensive delocalization in the initial diyne.

### Diazo Cycloadditions

**Fig. 23:**
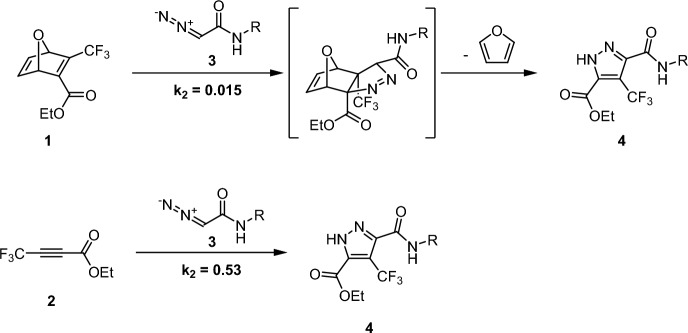
Reaction between oxanorbornadiene **1** and diazoacetamide 3 (top) and linear alkyne 2 and diazoacetamide 3 (bottom)

Diazo compounds serve as compelling 1,3-dipoles due to their enhanced reactivity compared to azides, while still showcasing commendable metabolic stability [83]. The Raines group conducted a study to evaluate the reactivity of both azides and diazoacetamides with oxonorbornadienes and linear alkynes [84]. They revealed that the rate constant for the diazo compound surpasses that of azides by approximately tenfold (0.015 M^−1^ s^−1^ for diazo compounds compared to 0.001 M^−1^ s^−1^ for azides [85], respectively). Notably, while azides react with oxanorbornadienes fivefold more rapidly than with alkynes [85], the diazo compound **3** demonstrates contrasting behavior. Its reaction with alkyne **2** is a staggering 35-fold faster than its reaction with oxanorbornadiene **1** (Fig. 23).

**Fig. 24:**
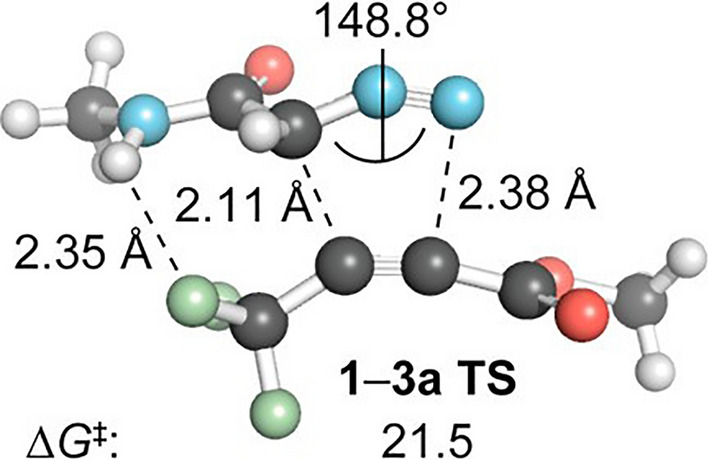
Optimized transition-state geometries for the cycloaddition of diazoacetamide **3** (R = Me) with alkyne **2** at the M06-2X/6–31 + G(2d,p) level of theory. Free energies (kcal/mol) include solvation corrections (MeOH) on gas-phase geometries with the IEFPCM model (radii = UFF).* IEFPCM* Integral equation formulation of the polarizable continuum model. Adapted with permission from [84]. Copyright 2016 American Chemical Society

The striking difference in reactivity prompted further exploration using computational techniques. The findings suggested that the reactivity of azides with strained alkenes is attributed to the ambiphilic nature of azides. This characteristic was emphasized by Sustmann in 1972, illustrating that azides exhibit increased reactivity with both electron-rich and electron-deficient alkenes [33, 86]. When the alkene double bond undergoes bending, the HOMO/LUMO gap is reduced [58], increasing normal and inverse electron demand orbital interactions during azide reactions. On the other hand, diazo dipoles, being predominantly nucleophilic, exhibit a strong preference towards electron-deficient dipolarophiles. As previously documented by Alabugin and coworkers, introducing a fluorine atom at the propargylic position of an alkyne induces hyperconjugation, rendering the alkyne more flexible and thereby reducing distortion energies. Moreover, interactions between the CF groups and the diazoacetamide amide hydrogen are present (Fig. 24), which further lowers the barrier, drawing parallels to phenomena observed in SPAAC.

The reaction between linear alkyne **2** (Fig. 23) and diazo compounds is highly asynchronous. In polar solvents, this is advantageous, as these solvents can help stabilize charges during the transition stage, increasing reactivity. Thus, solvent effects play a notable role in this reaction.

**Fig. 25:**
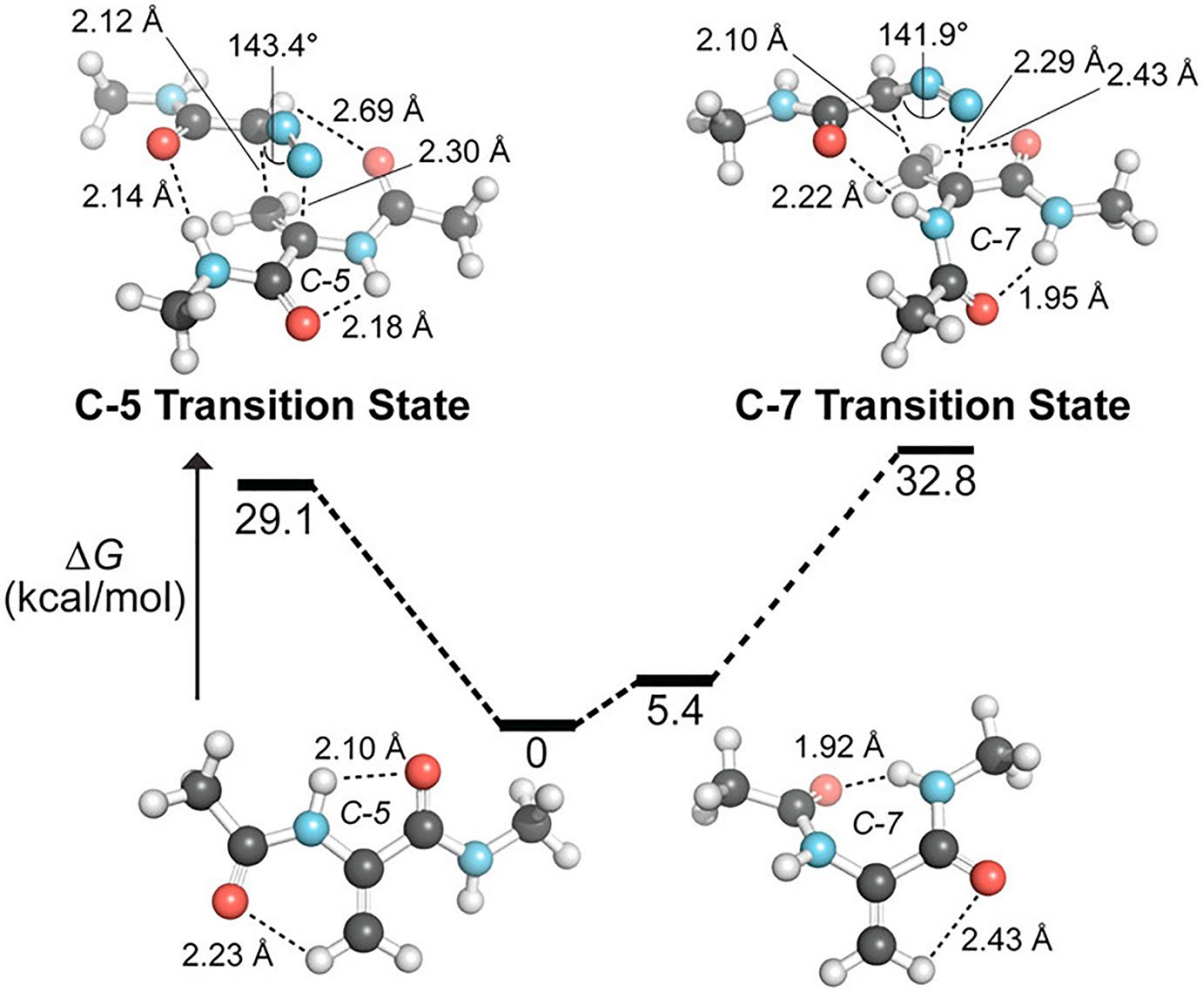
Computational analysis of the cycloaddition of 2-acetamido-N-methylacrylamide with N-methyl-2-diazoacetamide. Optimized geometries and free energies were calculated at the M06-2X/6–31 + G(2d,p) level of theory. Energies include solvation corrections (water) on gas-phase geometries using the IEFPCM model (radii = UFF). Reprinted with permission from [87]. Copyright 2016 American Chemical Society

Expanding on this, the Raines group proposed a method for the 1,3-dipolar cycloaddition of diazo compounds, even in the presence of azides [87]. The principle hinges on the need for strain to achieve satisfactory reaction rates with azides, but by tweaking the electronic properties, the reaction with diazo compounds can be enhanced. While acrylamides do selectively react with diazo compounds in the presence of azides, they might not be optimal for bioorthogonal reactions due to reaction with other biological compounds. However, it was observed that dehydroalanine can react with acceptable rates with diazoacetamides even when azides are present. Figure 25 depicts the transition states of 2-acetamido-N-methylacrylamide with N-methyl-2-diazoacetamide. Depending on the conformation of the dehydroalanine peptide, there are two possible transition states. In both scenarios, hydrogen bonding to the amide group of the diazoacetamide can be observed.

**Fig. 26:**
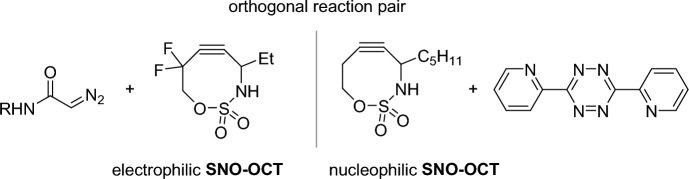
Orthogonal reaction pair based on **SNO-OCT** found by Schomaker and coworkers [88]

Schomaker, Raines, and coworkers showed that the **SNO-OCT** scaffold can be used to create an orthogonal pair of bioorthogonal reactions [88]. This cyclooctyne can be tuned to either react with 3,6-di(2-pyridyl)-1,2,4,5-tetrazines or diazoacetamides. Including exocyclic difluoro groups in the propargylic position, the electrophilicity of **SNO-OCT** can be increased to react rapidly with diazo compounds. Meanwhile electron-poor 3,6-di(2-pyridyl)-1,2,4,5-tetrazines reacts preferentially with electron-rich cyclooctynes. A computational screening revealed an orthogonal reaction pair (Fig. 26) which was confirmed experimentally.

### Nitrile Imine Cycloadditions

**Fig. 27:**
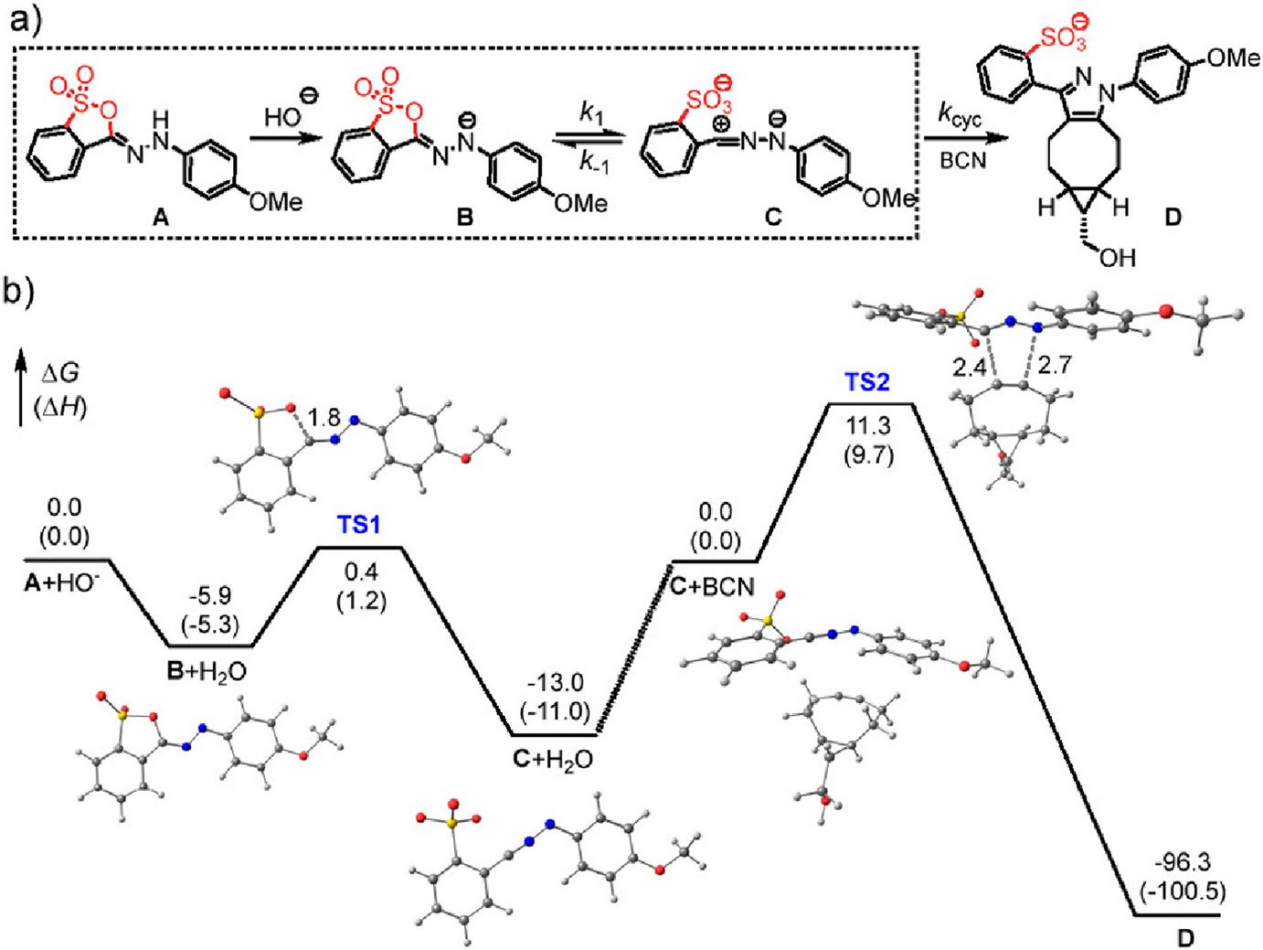
**a** Scheme of the reaction steps involved in HS → NI tautomerization (box) and subsequent 1,3-dipolar cycloaddition. **b** Reaction diagram showing the free energy profile involving HS-1 and the optimized structures. A, Neutral sultone form; B, anionic form of A; TS1, transition state for sultone ring rupture; C, nitrile imine form; C + BCN, reactant complex between C and BCN; TS2, transition state for the cycloaddition; D, cycloadduct. Energies are reported in kcal/mol, while interatomic distances at the transition states are reported in Å. Reprinted with permission from [89]. Copyright 2023 American Chemical Society

Nitrile imines are highly reactive, yet unstable 1,3-dipoles that often react with nucleophiles like water. As a result, they are typically generated in situ, for example through photolysis of 2,5-disubstituted tetrazoles. Lin and colleagues devised a way to effectively mask the nitrile imine, thus enhancing its stability [89]. Their approach involves transitioning hydrazonyl sultone **A** to its deprotonated form, **B**. This then establishes an equilibrium with the nitrile imine **C**, as depicted in Fig. 27. This 1,3-dipole is primed to rapidly react with strained alkynes, with **BCN** being a notable reaction partner. Computational insights emphasized the significance of the tautomerization and deprotonation processes. The findings also indicate that the equilibrium is sensitive to variations in pH and solvent polarity, a conclusion supported by experimental data.

### Cycloadditions of Mesoionic Compounds

**Fig. 28:**
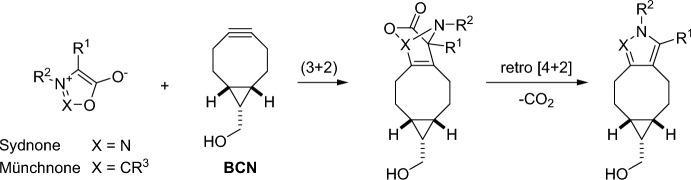
Reaction mechanism between mesoionic compounds and strained alkynes

Mesoionic compounds, like sydnones and münchnones, have made their mark in bioorthogonal chemistry due to their ability to react well with strained alkynes. This reaction proceeds through a (3 + 2) cycloaddition and is followed by a retro-Diels–Alder reaction that releases CO_2_ (Fig. 28) [90].

A study by Varnek and their team investigated the factors influencing the cycloaddition of sydnones with **BCN** [91]. These authors discerned that the size of the substituent R^1^ (Fig. 28) and the charge on the bond-forming carbon atom within the sydnone play crucial roles in determining reaction rates. Houk, Liang, and coworkers examined the implications of halogen substitutions, specifically the effects of fluorine, in bioorthogonal sydnone cycloadditions using DIA [92]. Their findings revealed that introducing a fluorine atom at the C4 position significantly decreases the activation barrier. This effect can be attributed to a combination of lowered LUMO energy and notably reduced distortion energy. The strong electron-withdrawing effect of the fluorine atom, when bonded to an sp^2^ hybridized carbon, effectively reduces the distortion energy, promoting the transition towards sp^3^ hybridization where the electron density can be more efficiently distributed in accordance with Bent's Rule [93–95].

**Fig. 29:**
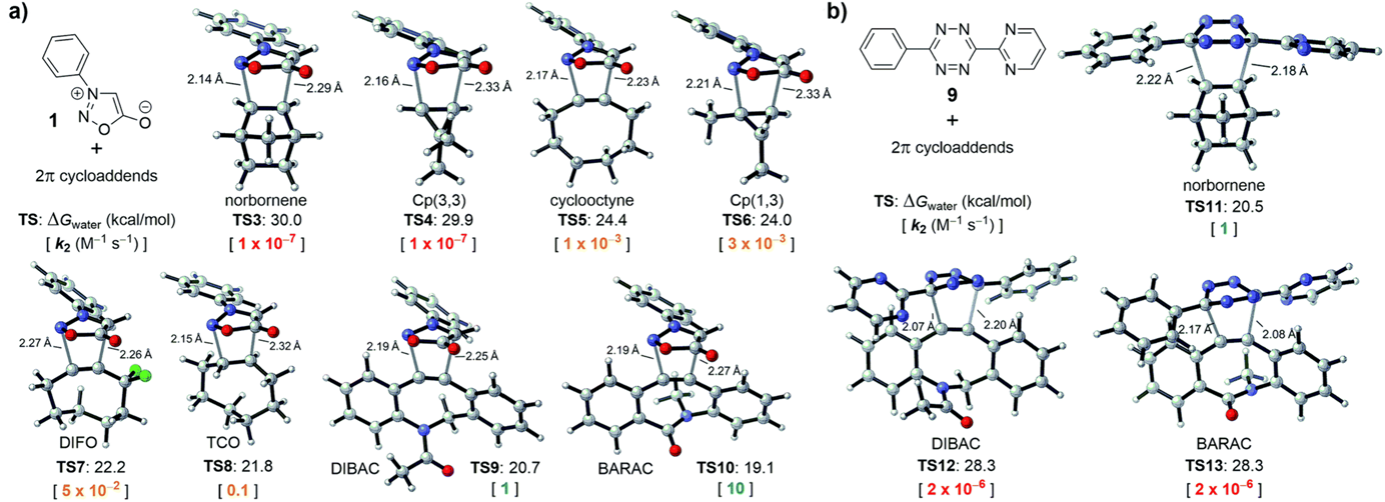
**a** Density functional theory-computed activation-free energies for the (3 + 2) cycloadditions of sydnone **1** with 8 strained alkenes and alkynes at the CPCM(water)-M06-2X/6–311 + G(d,p)//M06-2X/6-31G(d) level of theory and the predicted rate constants in water at 25 °C. **b** DFT-computed activation-free energies and predicted rate constants for the (4 + 2) cycloadditions of tetrazine **9** with norbornene, DIBAC, and **BARAC**. Republished with permission from [96]

Murphy, Houk, and coworkers combined DFT calculations with experimental work to explore and identify novel reactivities and selectivities related to bioorthogonal sydnone cycloadditions [96]. The computational data revealed that sydnones have a preference to react with dibenzocyclooctynes, as depicted in Fig. 29a. In contrast, the reactivity of diaryl-1,2,4,5-tetrazine towards these cyclooctynes is limited due to steric hindrance, making them more inclined to react with norbornenes. These predictions from the computational study were verified experimentally leading to an orthogonal click reaction pair.

**Fig. 30:**
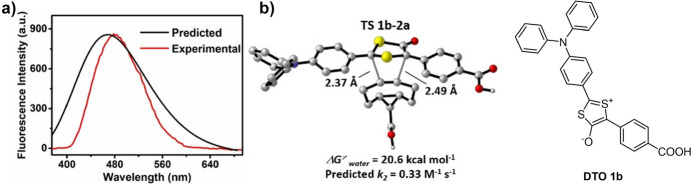
**a** Predicted spectrum of the click product of DTO 1b and **BCN** in water (black line) and experimental fluorescence spectrum (red line) in 5% DMSO in PBS (pH 7.4, 10 mM). **B** DFT-computed activation-free energy for the 1,3-dipolar cycloaddition between DTO 1b and **BCN** and predicted rate constant in water at 25 °C and structure of DTO 1b.* DMSO* Dimethyl sulfoxide, * DTO* 1,3-dithiolium-4-olates,* PBS* phosphate-buffered saline Adapted with permission from [98]

Taran and his team explored another category of mesoionic compounds, specifically 1,3-dithiolium-4-olates (DTOs). When these compounds react with cyclooctynes, they produce fluorescent thiophenes and release carbon sulfide [97]. Employing DFT calculations, the team anticipated the emission spectrum of the resulting product after the click reaction, as well as the reactivity of DTO 1b when exposed to **BCN**, as depicted in Fig. 30 [98]. The calculations pointed to a strong cycloaddition reactivity, a prediction that was subsequently validated through experimental work.

## [4 + 2] Cycloadditions

[4 + 2] Cycloadditions gained significant traction in bioorthogonal chemistry following the report of the tetrazine/*trans*-cyclooctene ligation in 2008 [99]. Since that milestone, a multitude of other [4 + 2] cycloadditions have emerged and these are also detailed in this chapter.

### 1,2,4,5-Tetrazine Cycloadditions

**Fig. 31:**
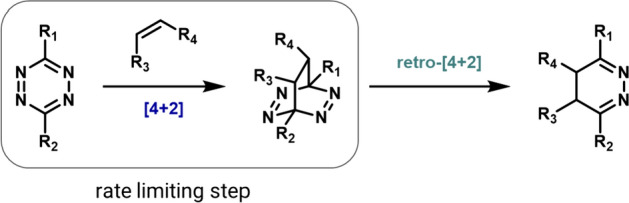
Mechanism of the tetrazine ligation

Since as far back as 1959, it has been established that 1,2,4,5-tetrazines can undergo inverse electron demand Diels–Alder cycloadditions with compatible dienophiles, followed by retro-cycloaddition that results in the loss of N_2_ (Fig. 31) [100]. It was in 1990 that Sauer demonstrated that strained alkenes, such as *trans*-cyclooctene (TCO), reacted especially well with 1,2,4,5-tetrazines [101]. However, the bioorthogonal nature of this reaction was not recognized until 2008 when it was documented by Fox and colleagues [99].

The high reactivity of 1,2,4,5-tetrazines in Diels–Alder reactions has been the subject of several computational studies.

**Fig. 32:**
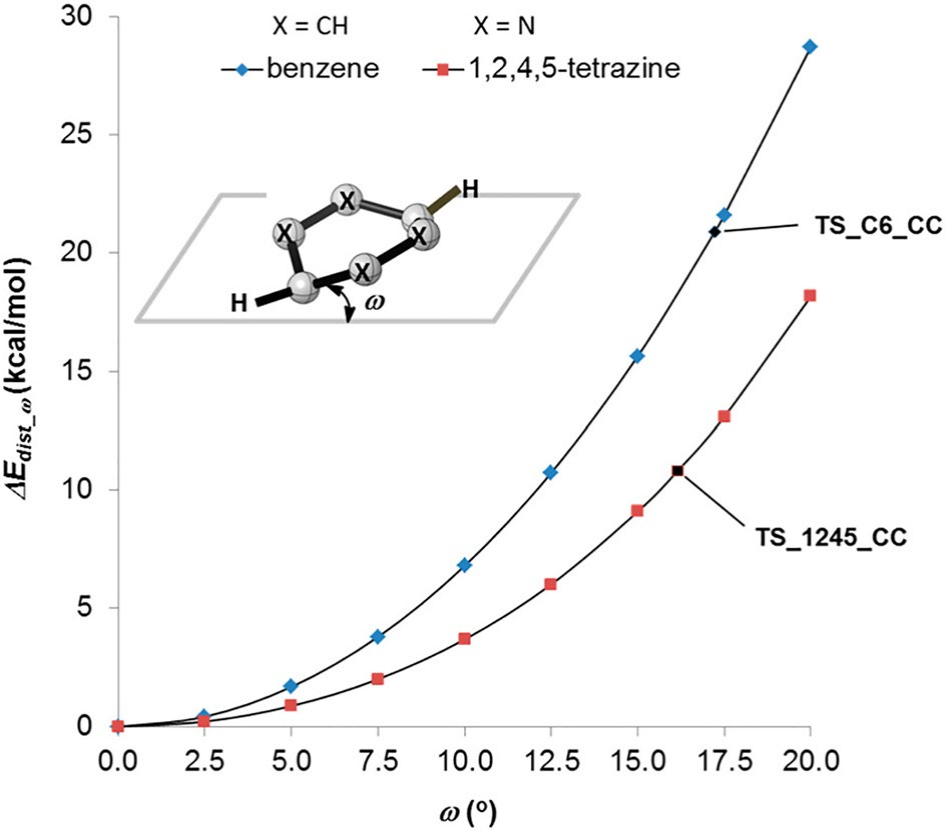
Plot of Δ*E*^‡^_dist_ω_ versus the out-of-plane dihedral angle ω for benzene and 1,2,4,5-tetrazine. Reprinted with permission from [102]. Copyright 2016 American Chemical Society

In 2016, Houk and his team studied various azines, including 1,2,4,5-tetrazines, using DIA [102]. They found that, compared to other azines like 1,2,4-triazine and pyridazine, 1,2,4,5-tetrazines have a lower distortion energy and an increased interaction energy. The lower distortion energy was linked to the reduced aromatic character of 1,2,4,5-tetrazines, making it easier for the necessary out-of-plane bending in the reaction. Figure 32 shows the difference in distortion energy between benzene and 1,2,4,5-tetrazine during this deformation.

Ess and his team investigated the increased interaction energies observed in a series of azines. They utilized energy decomposition analysis and pinpointed that reduced Pauli repulsion is the primary factor for the high reactivity [103].

**Fig. 33:**
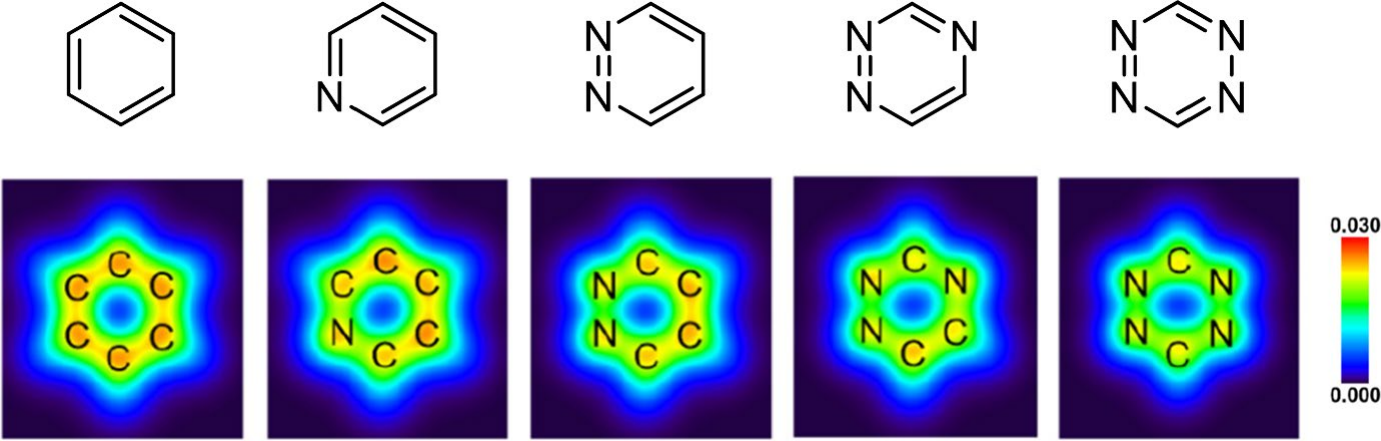
Electron density maps at 1.1 Å from the plane of benzene, azadienes, and 1,2,4,5-tetrazine. Adapted with permission from [30]. Copyright 2022 American Chemical Society

Houk and his group, in a comprehensive study on the frontier molecular orbital (FMO) model and energy decomposition of Diels–Alder cycloadditions, similarly highlighted that the high reactivity stems from reduced Pauli repulsion [30]. By examining the electron density at a typical bond formation radius (specifically half of the transition state distance between two carbon atoms in a typical Diels–Alder reaction) of 1.1 Å, these authors showed that sequentially adding nitrogen atoms lessens the electron density at the reactive carbons (Fig. 33). This reduction accounts for the decreased Pauli repulsion. Such visual insights serve as useful tools for creating qualitative models.

Similarly, by using conceptual DFT and molecular electron density theory as their analytical tools, Domingo and his team arrived at the same conclusion. They demonstrated that an increased number of nitrogen atoms in the ring leads to a reduced electron density and a consequent decrease in aromaticity. This change gives tetrazines a more electrophilic character, enhancing their reactivity [104].

#### Reaction with *trans*-Cyclooctenes

Houk and coworkers computationally examined the reactivity of tetrazines with *trans*-cyclooctenes [105]. Through DIA, they illustrated that the high reactivity of *trans*-cyclooctynes towards 1,2,4,5-tetrazines originates from a predistortion, leading to a decreased distortion energy.

**Fig. 34:**
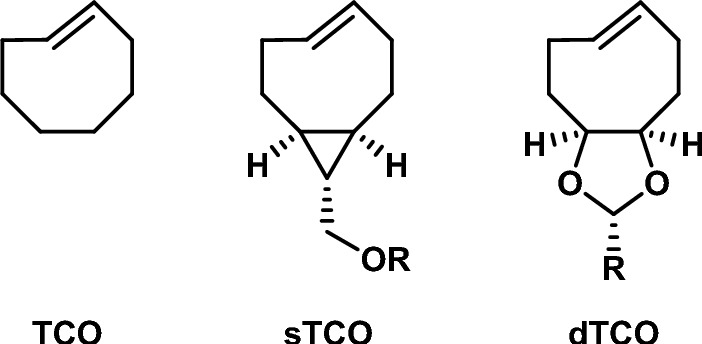
Commonly used *trans*-cyclooctene derivatives.* TCO* *trans*-Cyclooctene

In 2011, the Fox group introduced a more reactive derivative of *trans*-cyclooctene, termed **sTCO** (strained-TCO; Fig. 34) [106]. This was later followed by **dTCO** [107]. While **dTCO** shows reactivity between **sTCO** and TCO, it boasts better stability than **sTCO**. A commonality among these TCO derivatives is a* cis*-fused ring located opposite the double bond in the *trans*-cyclooctene.

**Fig. 35:**
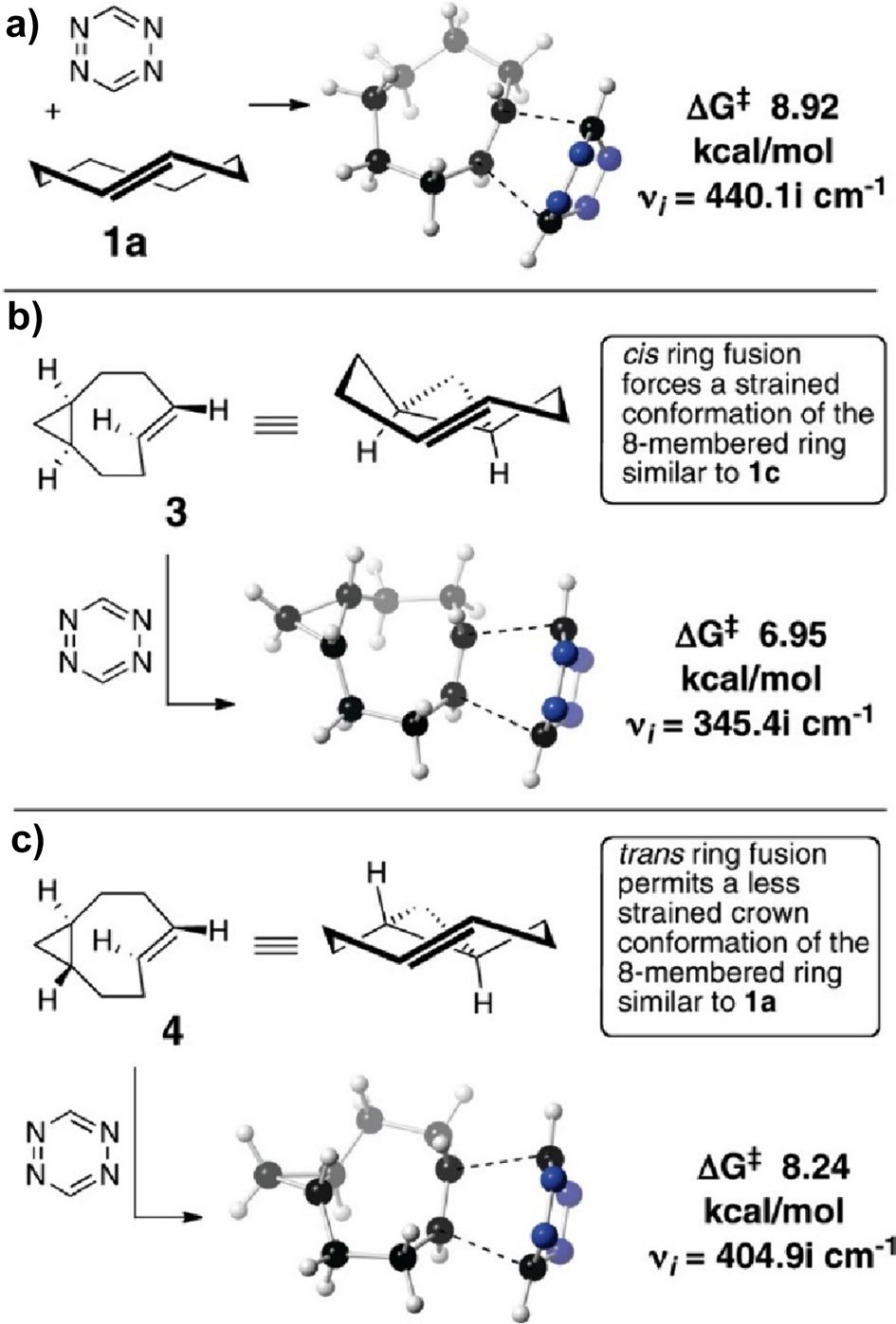
M06L/6–311 + G(d,p)-optimized transition structures for the Diels–Alder reaction of *s*-tetrazine with the crown conformer of *trans*-cyclooctene (**a**), the *cis*-ring fused bicyclo[6.1.0]non-4-ene **3** (**b**), and the *trans*-ring fused bicyclo[6.1.0]non-4-ene **4** (**c**). The barrier (8.24 kcal/mol) for the reaction of **4** with *s*-tetrazine is 1.29 kcal/mol higher than the analogous reaction of **3**. Reprinted with permission from [106]. Copyright 2011 American Chemical Society

Computational research showed that this* cis*-fused ring forces the TCO to assume a half-chair structure, rather than the crown conformation that is less strained and energetically favored. By forcing the molecule into this strained half-chair conformation, the energy barrier for the reaction is reduced (Fig. 35). Conversely, a *trans*-fused ring enables the molecule to adopt the crown conformation without increasing strain, thereby not significantly influencing reactivity.

**Fig. 36:**
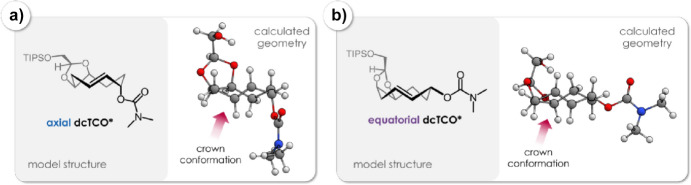
**a** Model structure ax-dcTCO*and its lowest energy conformer. **b** Model structure eq-dcTCO* and its lowest energy conformer. Reprinted with permission from [108]

Yet, when the *cis*-fused dioxolane ring is shifted one position, as seen in **dcTCO**, as recently presented by Mikula and team, it does not lead to a more strained crown conformation. This was confirmed through X-ray crystallography and a computational analysis (Fig. 36) [108].

Computational chemistry is frequently employed to predict reaction barriers for these click reactions prior to conducting experiments [109–111]. By examining reaction profiles, reactivities can be predicted. In 2017, Svatunek et al*.* explored the reactivity between *trans*-cyclooctene and ten distinct tetrazines. Utilizing experimental rate constants, they formulated a computational model that can estimate absolute reaction rates based on DFT-calculated barriers [112].

**Fig. 37:**
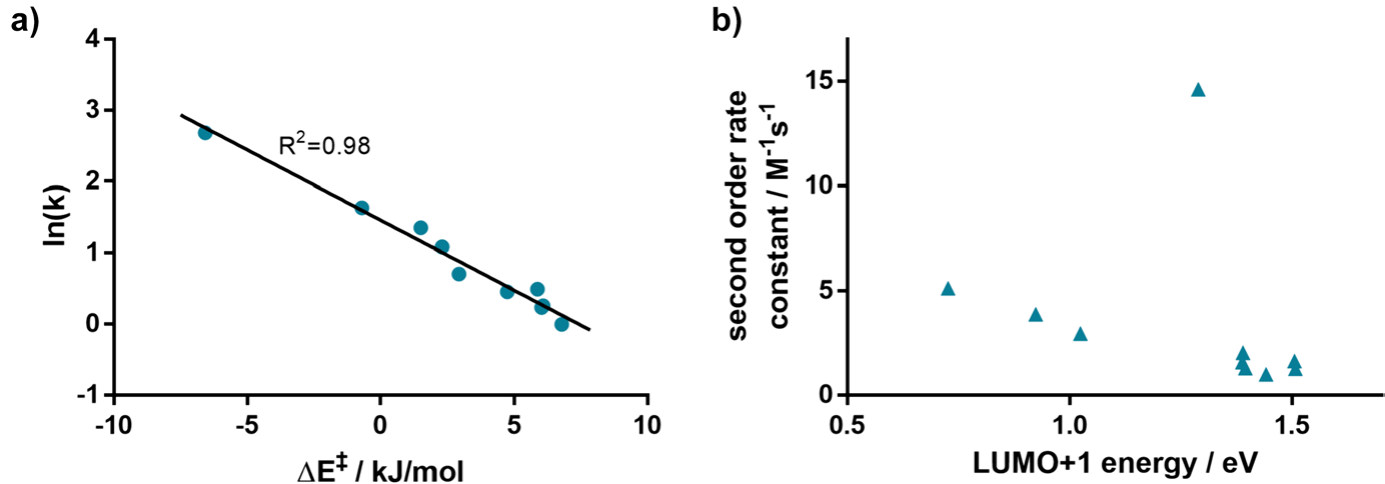
**a** Correlation between natural logarithm of second-order rate constants and M06-2X/6–311 + G(d,p) calculated energies of activation. **b** Second-order rate constants plotted against HF/6–311 + G(d,p)//M06-2X/6–311 + G(d,p) calculated LUMO+1 energies of the corresponding tetrazine. Reprinted with permission from Monatsh. Chem. 149:833–837 (2018) under the Creative Commons Attribution 4.0 International License [113]

Figure 37a displays the correlation between calculated ΔE^‡^ and ln(k). This correlation was utilized to derive a formula that allows for the prediction of second-order rate constants based on calculated ΔE^‡^. Interestingly, the study highlighted that the reactivity of the tetrazines under investigation did not align with the FMO theory (Fig. 37b). Specifically, 3-(pyrid-2-yl)-1,2,4,5-tetrazine exhibited a notable deviation and showcased markedly enhanced reactivity than what FMO theory would predict.

**Fig. 38:**
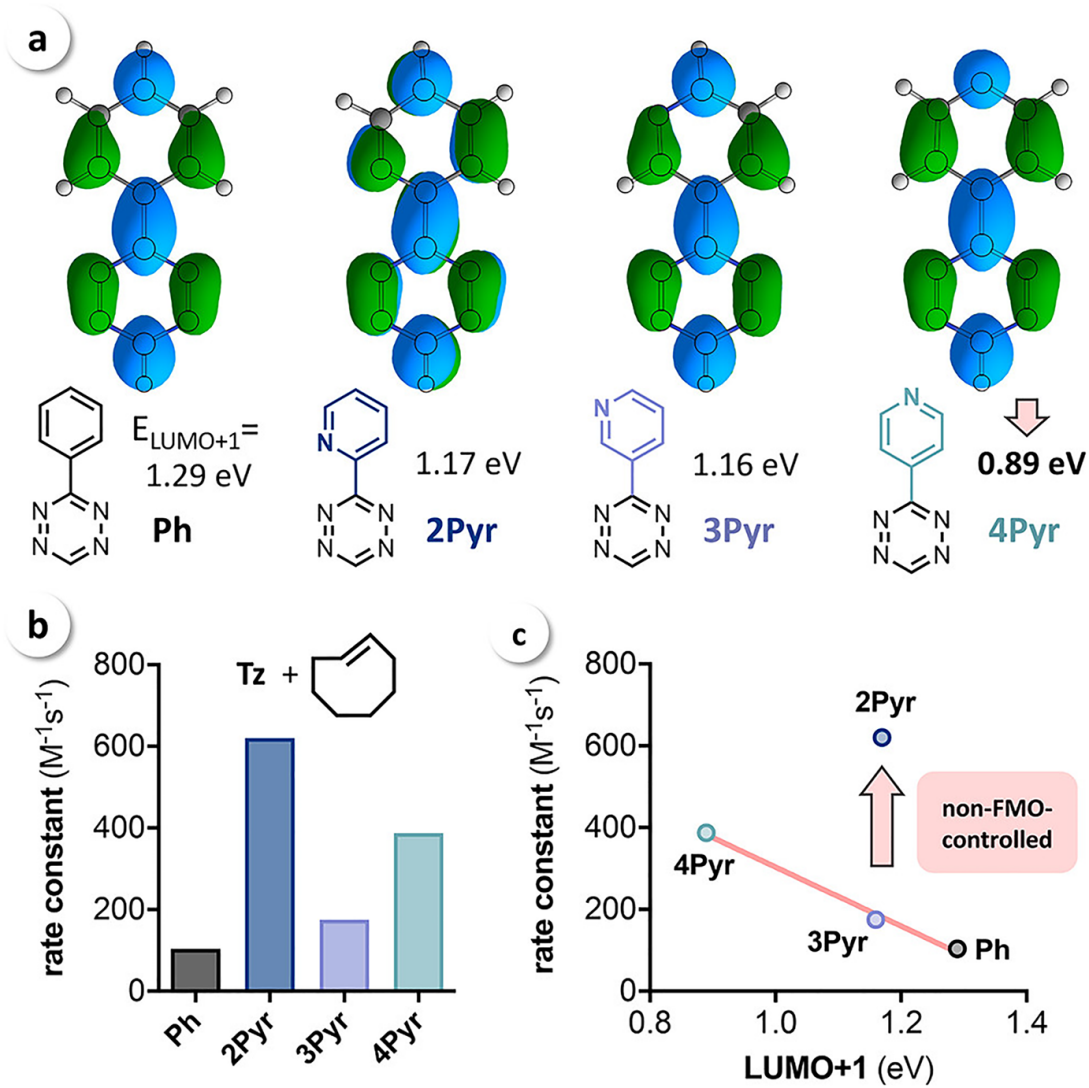
**a** LUMO+1 orbitals and orbital energies of mono-substituted Tz showing a significantly lower LUMO+1 energy for **4Pyr**; **b** rate constants for the reaction of Ph, **2Pyr**, **3Pyr**, and **4Pyr** with TCO (1,4-dioxane, 25 °C, *n* = 6, SD < 1%); **c** measured rate constants vs calculated LUMO+1 energy.* Ph* Phenyl-tetrazine,*2Pyr, 3Pyr, 4Pyr* isomers 2-pyridyl, 3-pyridyl, 4-pyridyl, respectively, * Tz* tetrazines. Reprinted with permission from [114]

Intrigued by this unusual reactivity of 2-pyridyl tetrazines, Svatunek et al*.* investigated pyridyl tetrazines [114]. Through a combined approach of experimental and computational methods, they assessed the reactivity of the isomers 2-pyridyl (**2Pyr**), 3-pyridyl (**3Pyr**), and 4-pyridyl (**4Pyr**), along with phenyl-tetrazine (Ph) as shown in Fig. 38. The findings revealed that these tetrazines did not adhere to FMO theory. Remarkably, **2Pyr** emerged as more reactive than its counterparts, even with its elevated LUMO+1 energy.

**Fig. 39:**
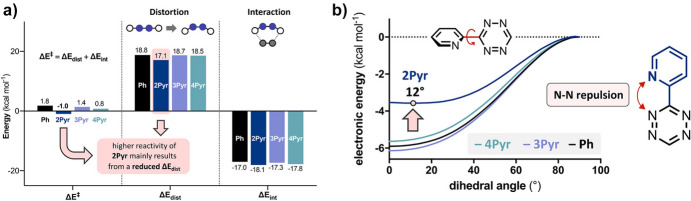
**a** Distortion/interaction analysis shows that the high reactivity of 2-pyridyl-substituted Tz results from a reduced distortion energy (Δ*E*_dist_). **b** Calculated energy profiles for the rotation of the aryl–Tz bond showing that N–N repulsion reduces the stabilization energy for **2Pyr**. Adapted with permission from [114]

DIA revealed a notably reduced distortion energy for **2Pyr**, as depicted in Fig. 39a. The underlying reason for this was identified as a destabilizing N–N lone pair repulsion present in the reactant. This repulsion reduces the barrier for rotation, consequently reducing the distortion energy, as illustrated in Fig. 39b. This discovery carries profound importance as it allows for high reactivity without compromising stability, a notion that the authors have experimentally corroborated.

**Fig. 40:**
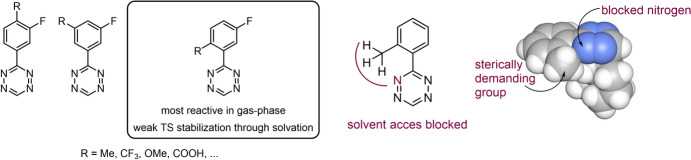
Ortho-, meta-, and para-substituted tetrazines investigated by Battisti et al*.* and visualization of sterically hindrance of solvent access.* TS* Transition state

It can be suggested that other substituents causing predistortion between an aryl group and a tetrazine would result in decreased distortion energy, thereby speeding up the reaction. Battisti et al*.* explored mono-aryl tetrazines with substituents positioned ortho, meta, and para relative to the tetrazine (Fig. 38) [115]. Computational analyses in the gas phase indicated that substituents in the ortho position indeed increase the reactivity when compared to substituents in the meta or para positions. However, this computational prediction did not align with experimental observations. The discrepancy was attributed to solvent effects. Diels–Alder reactions are frequently accelerated by polar solvents. As the reaction progresses, charge transfer leads to a more polar transition state, which is stabilized more by solvents than the reactants are, thereby reducing the energy barrier. When utilizing implicit solvent models for calculations, it was revealed that the solvent effects are substantially less pronounced for compounds bearing ortho substituents. This can be explained by the fact that the ortho substituent acts as a shield, blocking the solvent from effectively stabilizing the tetrazine (Fig. 40).

Houszka et al. conducted a thorough investigation into the effects of tetrazine substituents [116]. This study not only looked at the commonly used substituents, such as alkyl, aryl, and hydrogen, but also provided insights into the increased reactivity of mono-substituted tetrazines. For example, 3-phenyl-1,2,4,5-tetrazine displayed greater reactivity than its 3,6-diphenyl-1,2,4,5-tetrazine counterpart. Through the application of DIA and EDA, it was demonstrated that the side without a substituent exhibited reduced Pauli (steric) repulsion. This allows the tetrazine to adopt an asynchronous approach. Although this reduces orbital overlap, it significantly reduces both distortion energy and Pauli repulsion, leading to high reactivity.

**Fig. 41:**
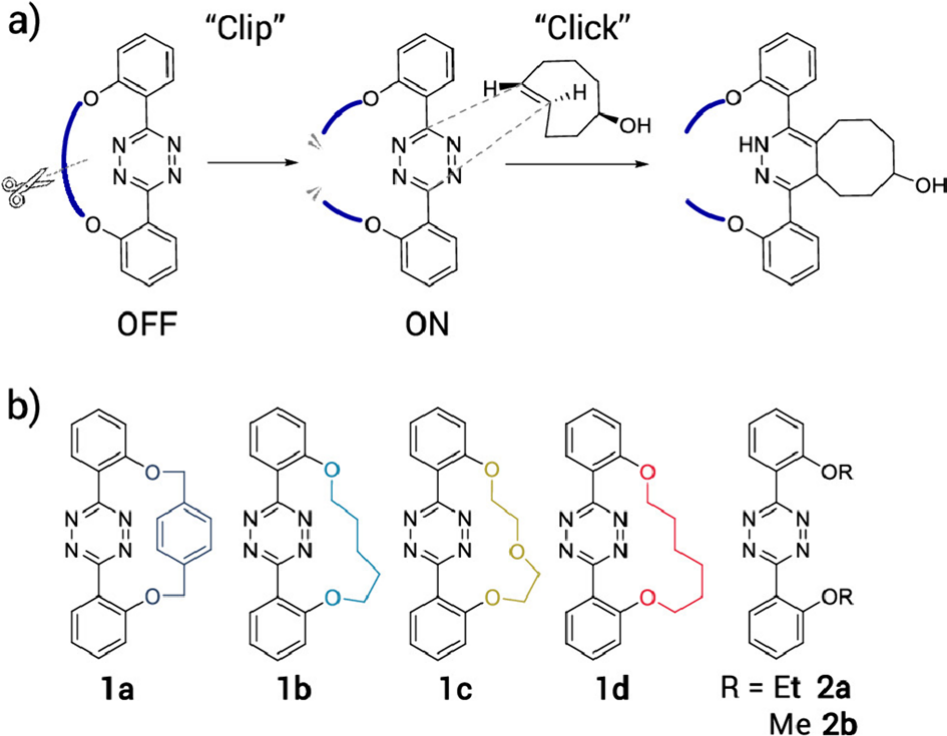
**a** “Clip to Click” system for controlling the IEDDA reaction of tetrazines and TCO. **b** Structures of used macrocyclic and acyclic tetrazines. Reprinted with permission from [117]. Copyright 2022 American Chemical Society

Mizukami and colleagues introduced a unique tetrazine reactant in their study. Their approach involved the use of macrocyclic tetrazines that remained less reactive with *trans*-cyclooctenes until a tether was removed (Fig. 41) [117]. This approach was aptly named 'clip to click'.

**Fig. 42:**
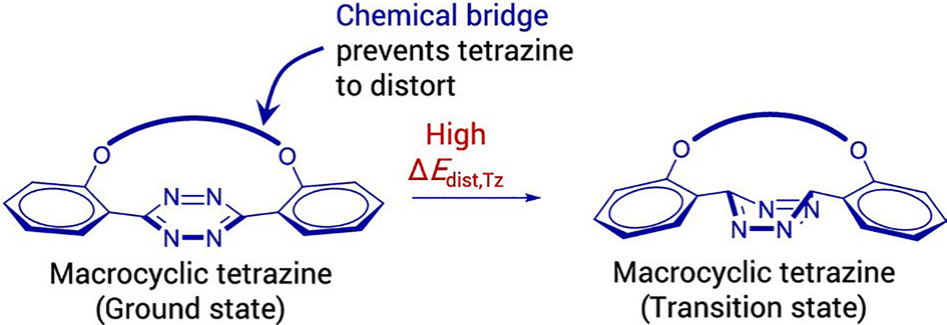
DIA showed that the high activation energy of macrocyclic tetrazines mainly results from the high distortion energy of tetrazine (Δ*E*_dist,Tz_). Adapted with permission from [117]. Copyright 2022 American Chemical Society

Through a computational examination utilizing DIA, it was determined that the bridged tetrazine had significantly elevated distortion energies, resulting in slower reactions (Fig. 42).

**Fig. 43:**
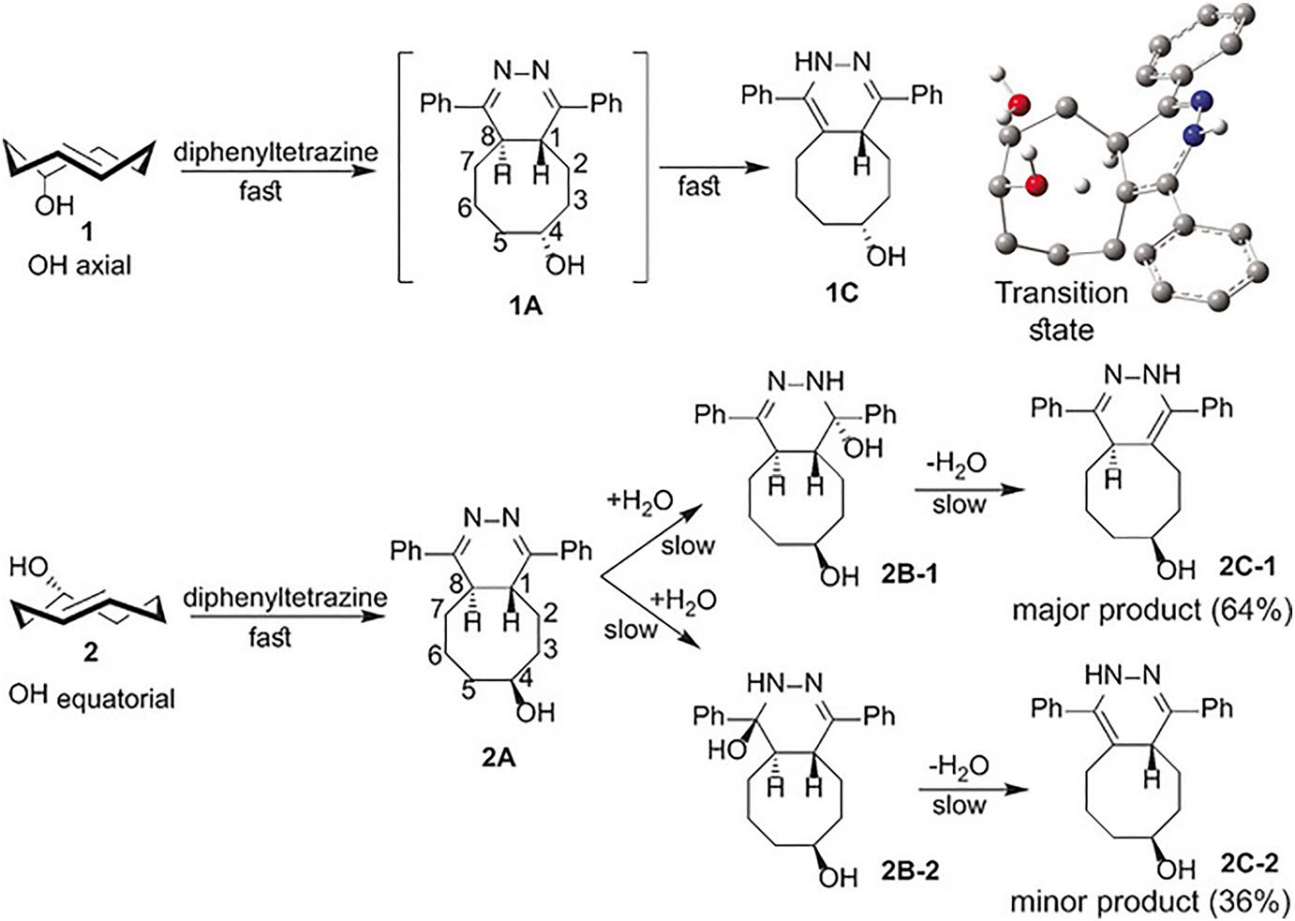
The product distribution arising from the reaction of diphenyltetrazine with the TCOs **1** and **2** as confirmed by nuclear magnetic resonance (NMR) analysis. Upper right: the transition-state connecting **1 A** and **1 C** (N-protonated form). Reprinted from [118]

In 2016, Vrabel and colleagues demonstrated that the two potential diastereomers of the 5-hydroxy derivative of TCO exhibited markedly different behaviors when reacted with fluorogenic tetrazines [118]. Upon undergoing a Diels–Alder reaction followed by N_2_ elimination, the resulting dihydropyridazines can exhibit fluorescence. Interestingly, only the reaction product of one TCO diastereomer displayed this fluorescence. Through a combination of experimental and computational methods, the authors were able to elucidate that an axial hydroxy group on the cyclooctene facilitates fast tautomerization via transannular proton transfer, leading to a fluorescent tautomer. Conversely, when the TCO has an equatorial hydroxy group, this tautomerization process is slow and no fluorescence is present, as depicted in Fig. 43.

Liu and colleagues showed that the reactivity of tetrazine can be reversibly modulated by enclosing the tetrazine within a guest–host system [119]. Specifically, in the presence of a naphthotube, a 3-methyl-6-phenyl-1,2,4,5-tetrazine reacts three orders of magnitude more slowly than in its absence. This property can be exploited to selectively shield tetrazines on a biomolecule from reacting with TCO. Meanwhile, unshielded tetrazines will readily react. Once the host is removed, the previously shielded tetrazines can engage in reactions in a subsequent step. To validate this strategy, the authors aimed to selectively react some tetrazines on a green fluorescent protein while masking others. To determine which tetrazines are accessible for interaction with naphthotubes and can therefore be shielded, the authors utilized computational docking studies.

#### Reaction with Cyclopropenes

**Fig. 44:**
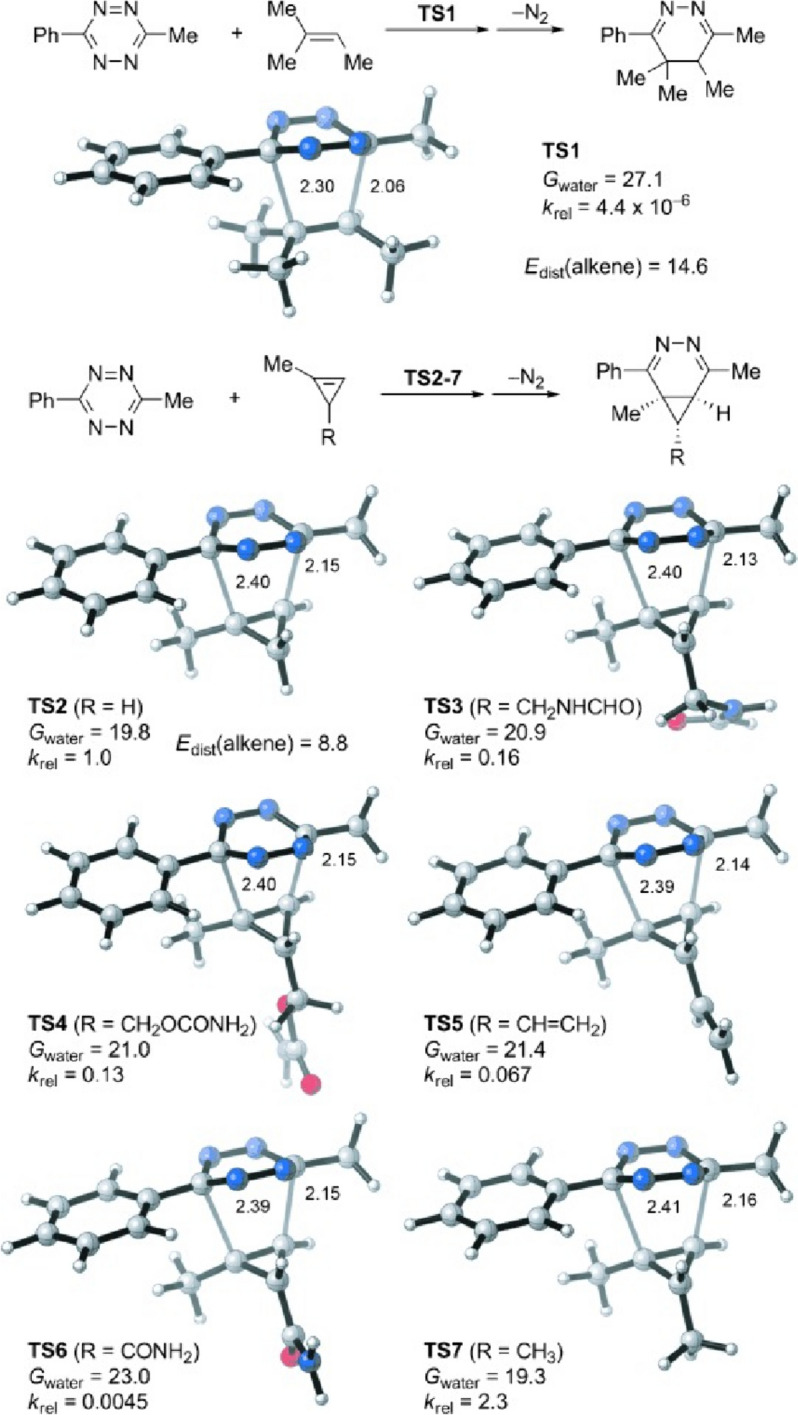
M06-2X/6-31G(d)-optimized transition-state structures for the Diels–Alder reactions of 3-methyl-6-phenyltetrazine with 2-methyl-2-butene and six methylcyclopropenes (distances in Å) and M06-2X/6–311 + G(d,p)//6-31G(d)-computed activation-free energies in water (*G*_water_, in kcal mol^−1^) and relative rate constants (*k*_rel_). Reprinted with permission from [121]

Other dienophiles that can be employed in bioorthogonal cycloadditions with tetrazines are cyclopropenes. Owing to their small size, they have been labeled as “mini-tags”. Nevertheless, their reactivity is markedly less than that of *trans*-cyclooctenes [120]. Houk, Devaraj, and coworkers conducted an in-depth investigation of the reactivity of these bioorthogonal reactants, employing both computational and experimental methods [121]. Initially, they compared the reactivity of 1-methyl-cyclopropene with the unstrained 2-methyl-2-butene. Despite the elevated HOMO energy in the linear alkene, cyclopropane exhibited greater reactivity (Fig. 44). This reactivity, not governed by FMO interactions, can be attributed to a reduced distortion energy for the cyclopropene, making it more amenable to distortion into the transition state. Following this, they looked into the effects of different substituents at the C3 position. Their findings revealed that, within the series of 1-methyl-cyclopropenes, HOMO energies are pivotal in determining reactivity.

In 2015, K. N. Houk celebrated the publication of his 1000th paper, entitled "Molecular dynamics of the Diels–Alder reactions of tetrazines with alkenes and N_2_ extrusions from adducts" [122]. In this study, Houk and his team investigated the reaction between 1,2,4,5-tetrazine and cyclopropene using quantum chemistry-level molecular dynamics. Their primary focus was determining whether the N_2_ elimination from the initial Diels–Alder product occurred in the anti or syn position relative to the cyclopropane ring of the initial Diels–Alder adduct. Transition state theory suggested an anti-elimination preference by around 9 kcal/mol. Contrarily, dynamic simulations pointed to a product ratio of 80% syn to 20% anti. This discrepancy was attributed to vibrational coupling. After the initial cycloaddition, the resultant intermediate holds excess vibrational energy, which may transition to a mode linked with *anti*-elimination. Fascinatingly, the intermediate cycloaddition product's lifetimes from dynamic calculations were notably shorter than those predicted by transition state theory. In numerous trajectories, the system essentially bypassed this intermediate in a duration shorter than a standard C–C stretch vibration, indicating strong non-statistical behavior.

#### Reaction with Isonitriles

**Fig. 45:**
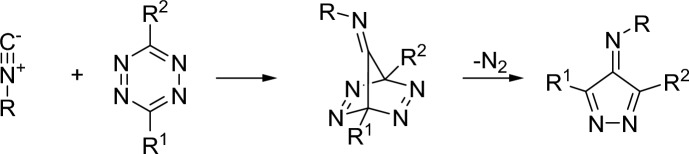
Reaction between isonitriles and tetrazines

Isonitriles react in a bioorthogonal manner with 1,2,4,5-tetrazines in a (1 + 4) cycloaddition, followed by a cycloreversion with loss of dinitrogen (Fig. 45) [123–125]. This reaction can also be modified to release leaving groups [126, 127].

**Fig. 46:**
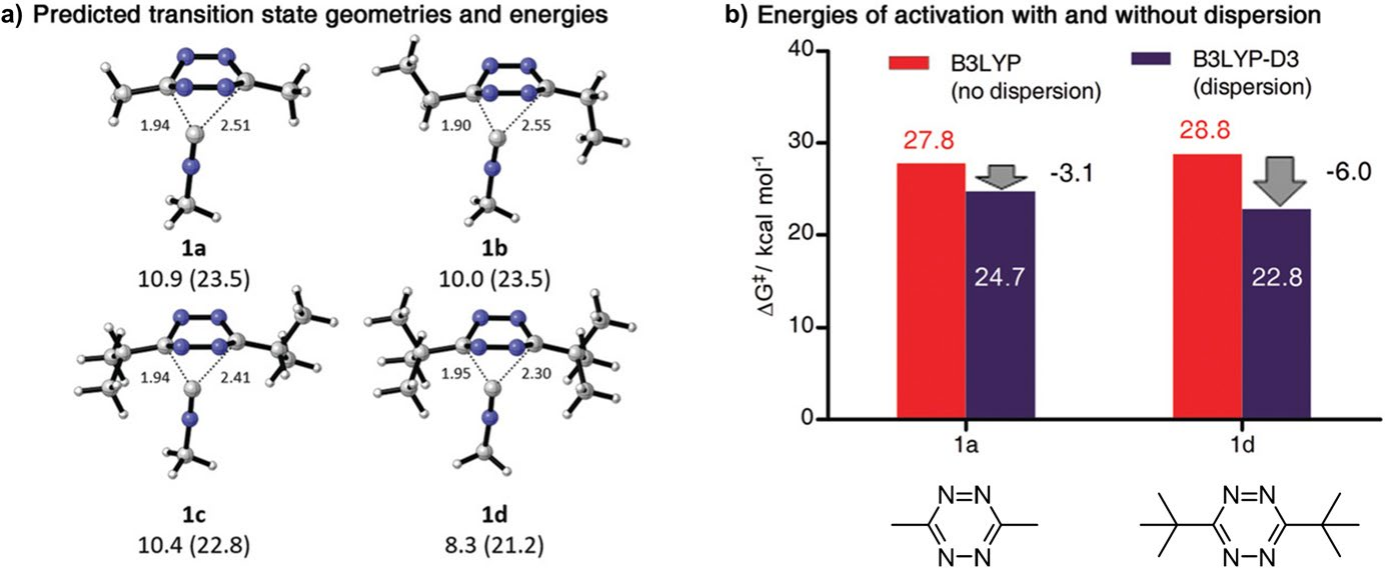
Dispersion forces increase the reactivity of isonitriles to tetrazines with bulky substituents. **a** M06-2X-D3(SMD) calculated transition state geometries and energies of activation for the rate-determining step of the reaction of tetrazines **1 a**–**d** with methylisonitrile. Energies are in kcal mol^−1^, distances are in Å, electronic energy, and Gibbs free energy (in brackets) is shown. **b** Gibbs free energies of activation (kcal mol^−1^) for the reaction of methylisonitrile and tetrazines **1 a** and **1 d** were calculated using the B3LYP functional with and without correction for dispersion forces. Adapted with permission from [128]

In 2019, the teams of Franzini and Houk observed that tetrazines with bulkier substituents, such as tert-butyl groups, exhibited reduced reactivity towards alkenes like norbornene due to steric hindrance. However, these same tetrazines displayed increased reactivity towards isonitriles [128]. A computational study attributed this rise in reactivity to favorable dispersion interactions between the bulky tetrazine substituents and the compact, linear isonitrile, which seems to fit well between the larger groups. Conversely, the larger alkenes appeared to be sterically blocked. The computational study made comparisons between results with and without dispersion corrections, highlighting the significance of dispersion in these reactions, as depicted in Fig. 46.

**Fig. 47:**
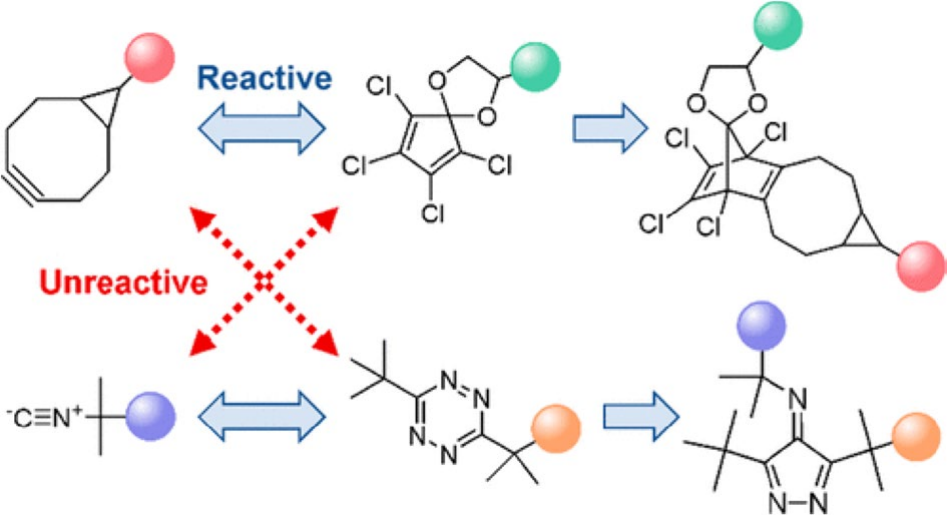
Orthogonality between isonitrile/tetrazine and BCN/TCK cycloadditions.* TCK* Tetrachlorocyclopentadienone ethylene ketal Reprinted with permission from [129]

Expanding on this understanding, Svatunek et al. demonstrated that while isonitriles quickly react with tetrazines that have significant steric demands, they remain unreactive with dienes like tetrachlorocyclopentadienone ethylene ketal (TCK) or 4,4-difluoro-3,5-diphenyl-4H-pyrazole (DFP) [129]. The reactivity of these dienes with strained alkynes is discussed in section 4.3. The computational analysis indicated that isonitriles remain unreactive with TCK or DFP due to a substantial HOMO/LUMO gap, resulting in low interaction energy. Since isonitriles do not engage with five-membered cyclic dienes and cyclooctynes do not react with tetrazines having bulky substituents because of steric constraints, a set of two independent bioorthogonal click reactions that do not interfere with each other was established (Fig. 47).

#### Reaction with Norbornene and Norbornene Derivatives

Around the same time as the emergence of the* trans*-cyclooctene tetrazine click chemistry, Hildebrand and coworkers introduced the use of the norbornene tetrazine for bioorthogonal antibody modification [130]. Despite its potential, this cycloaddition has garnered less attention, largely due to its reduced reactivity compared to the* trans*-cyclooctene tetrazine system. However, it still presents unique reactivity patterns that could be valuable in specific applications.

**Fig. 48:**
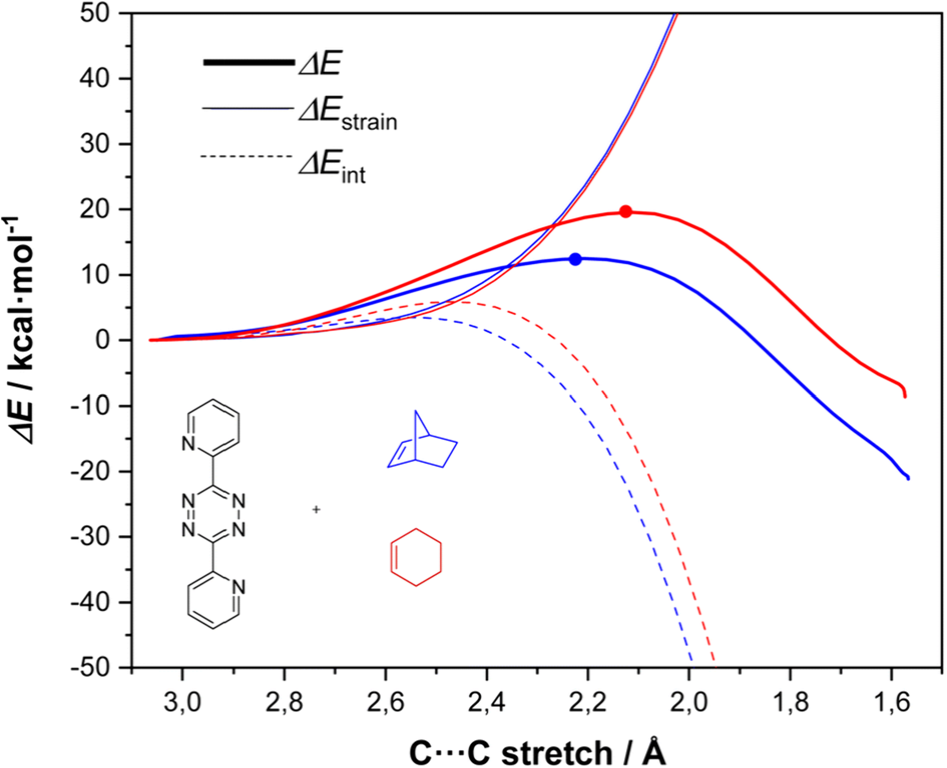
Activation strain analysis of the IEDDA of 3,6-dipyridin-2-yl-1,2,4,5-tetrazine with norbornene (blue) or cyclohexene (red) along the reaction coordinate projected onto the C···C bond stretch, computed at M06-2X/6–311 + G(d,p). The TS is indicated with a filled circle. Reprinted with permission from [131]

García-Aznara and Escorihuela explored the reaction through computational means [131]. Employing DIA across a reaction coordinate, they demonstrated that the superior reactivity of norbornene compared to cyclohexene stems from enhanced interaction energy rather than a decrease in strain energy (Fig. 48). A higher HOMO in case of norbornene was identified as the cause. Moreover, the authors examined the impact of norbornene substituents.

Klingler and Holland investigated the effects of tetrazine protonation on the reactivity of the click reaction, concluding that it has minimal impact on the rate-determining initial step [132].

**Fig. 49:**
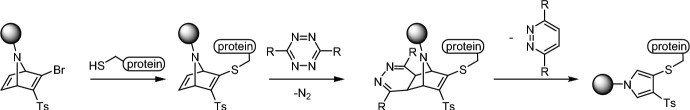
Synthetic strategy for a stable cysteine modification developed by Bernardes and coworkers[133]

Bernardes and colleagues innovated a system using azanorbornadienes to covalently modify cysteine, resulting in a stable bond [133]. Typically, cysteine is modified through the Michael addition with polarized double bonds, but such reactions tend to be reversible, causing the final adduct to have reduced stability [134]. By employing azanorbornadiene bromides as electrophiles, cysteine first gets modified with an azanorbornadiene. After this reacts with a tetrazine, a retro-Diels–Alder reaction occurs, resulting in a stable pyrrol linkage (as depicted in Fig. 49). Computational assessments initially confirmed the feasibility of this approach, after which it was effectively demonstrated experimentally.

**Fig. 50:**
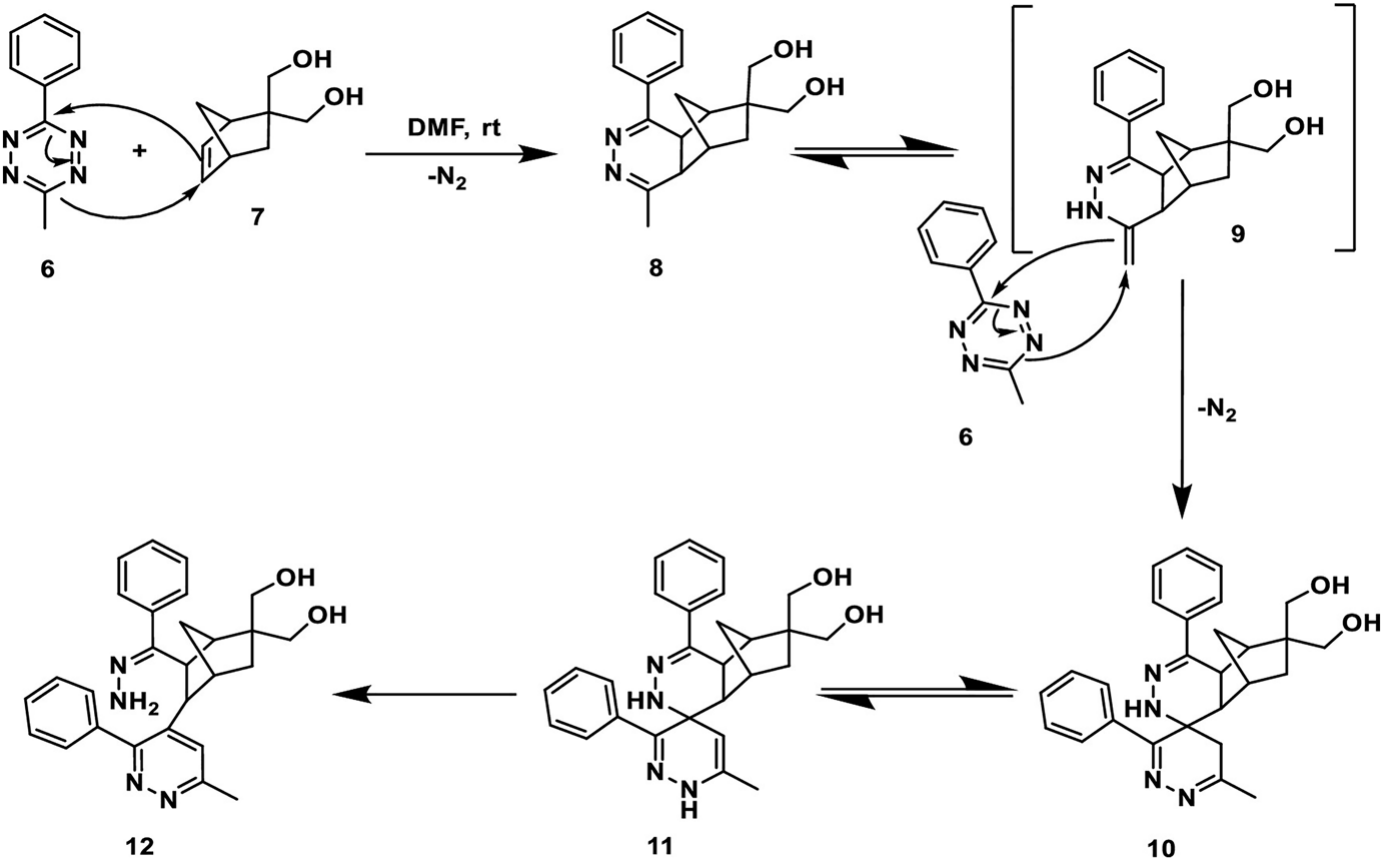
Plausible mechanistic pathway of 1:2 reactivity between 5-norbornene-2,2-dimethanol (**7**) and 3-methyl-6-phenyl-1,2,4,5-tetrazine (**6**). Reprinted with permission from [135]. Copyright 2023 American Chemical Society

Watts and coworkers noted that specific tetrazine derivatives display a reaction with a 2:1 ratio of tetrazine to norbornene, deviating from the conventional 1:1 ratio [135]. The researchers observed that one norbornene molecule reacts with two molecules of 3-aryl-6-methyl-1,2,4,5-tetrazines. A suggested mechanism for this pathway is illustrated in Fig. 50. Following the primary Diels–Alder and subsequent retro-Diels–Alder reactions, the imine double bond has the potential to shift its position via tautomerization to the exocyclic position. This newly formed alkene can then partake as a dienophile in another click reaction with an additional tetrazine. This proposition aligns with results from DFT computational studies performed by the authors.

**Fig. 51:**
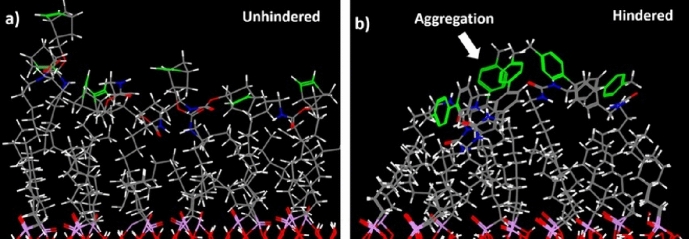
Comparison of surface disposition of IEDDA reactants in “free” ME after molecular dynamics of norbornene_(surf.)_ (**a**) and tetrazine_(surf.)_ (**b**). Reprinted with permission from [136]

Zuilhof and coworkers employed the tetrazine/norbornene ligation method for surface modification [136]. Notably, they observed that when surfaces were treated with norbornene and then reacted with tetrazine in a solution, the reaction rate was approximately double compared to when surfaces were treated with tetrazine and subsequently reacted with free norbornene. This observed difference was ascribed to two distinct reasons. Firstly, molecular dynamics simulations revealed that tetrazines tend to clump together via π–π stacking interactions, which restricts the number of tetrazine sites that are available for reaction (as depicted in Fig. 51). Secondly, the orientation of the reaction partners relative to the surface plays a significant role. When the surface is modified with norbornene, the tetrazine must approach it vertically from above. In contrast, when the surface is modified with tetrazine, norbornene must approach it laterally, almost parallel to the surface. This latter approach poses more steric challenges, making the reaction less efficient.

#### Reaction with Vinylboronic Acids

**Fig. 52:**
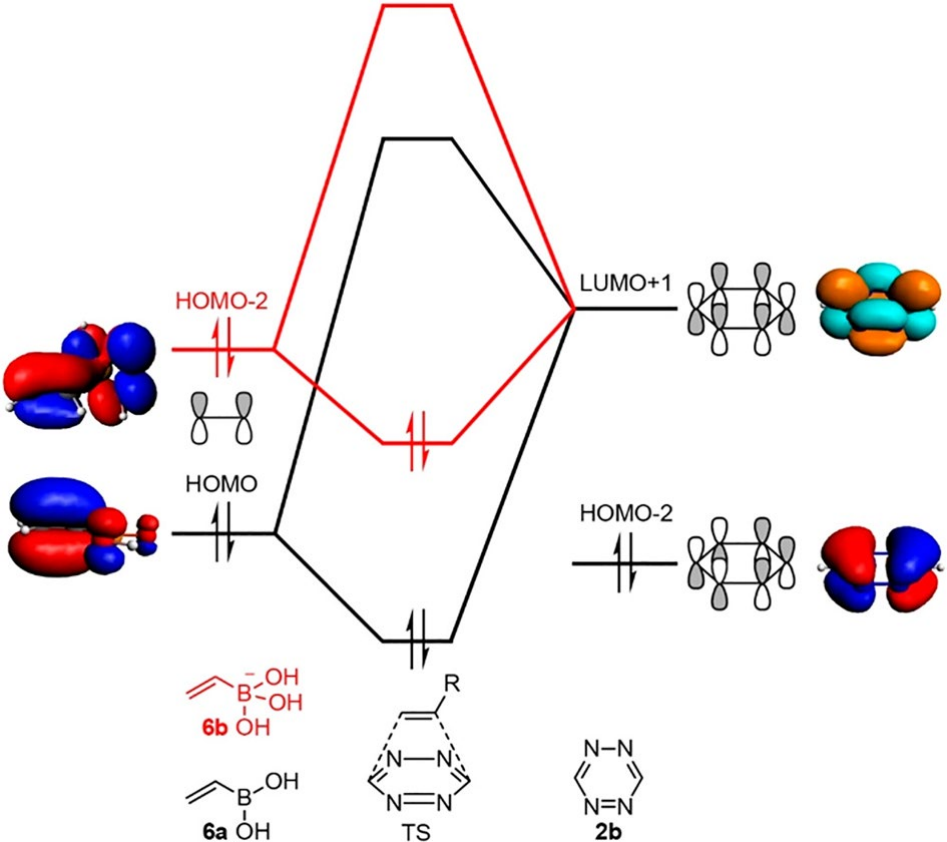
FMO in the tetrazine ligation. Schematic representation of the FMO interactions of 1,2,4,5-tetrazine **2b** and vinylboronic acid **6a** (black) or vinylboronate **6b** (red) from our quantitative Kohn–Sham MO analyses.* FMO* Frontier molecular orbital Reprinted with permission from [137]

Bonger, Bickelhaupt, and coworkers investigated the coordination-assisted bioorthogonal ligation between 2-phenyl tetrazines and vinylboronic acids [137]. The reaction is considerably sped up when a vinyl boronic acid coordinates with a 2-phenyl substituted tetrazine, resulting in a vinylboronate. This acceleration is primarily attributed to enhanced orbital interaction, a consequence of the more electron-rich and, consequently, elevated HOMO of the negatively charged vinylboronate (Fig. 52). Hence, these relatively electron-rich tetrazines exhibit a high selectivity and reactivity towards vinyl boronic acids.

#### Reaction with Cyclooctynes

**Fig. 53:**
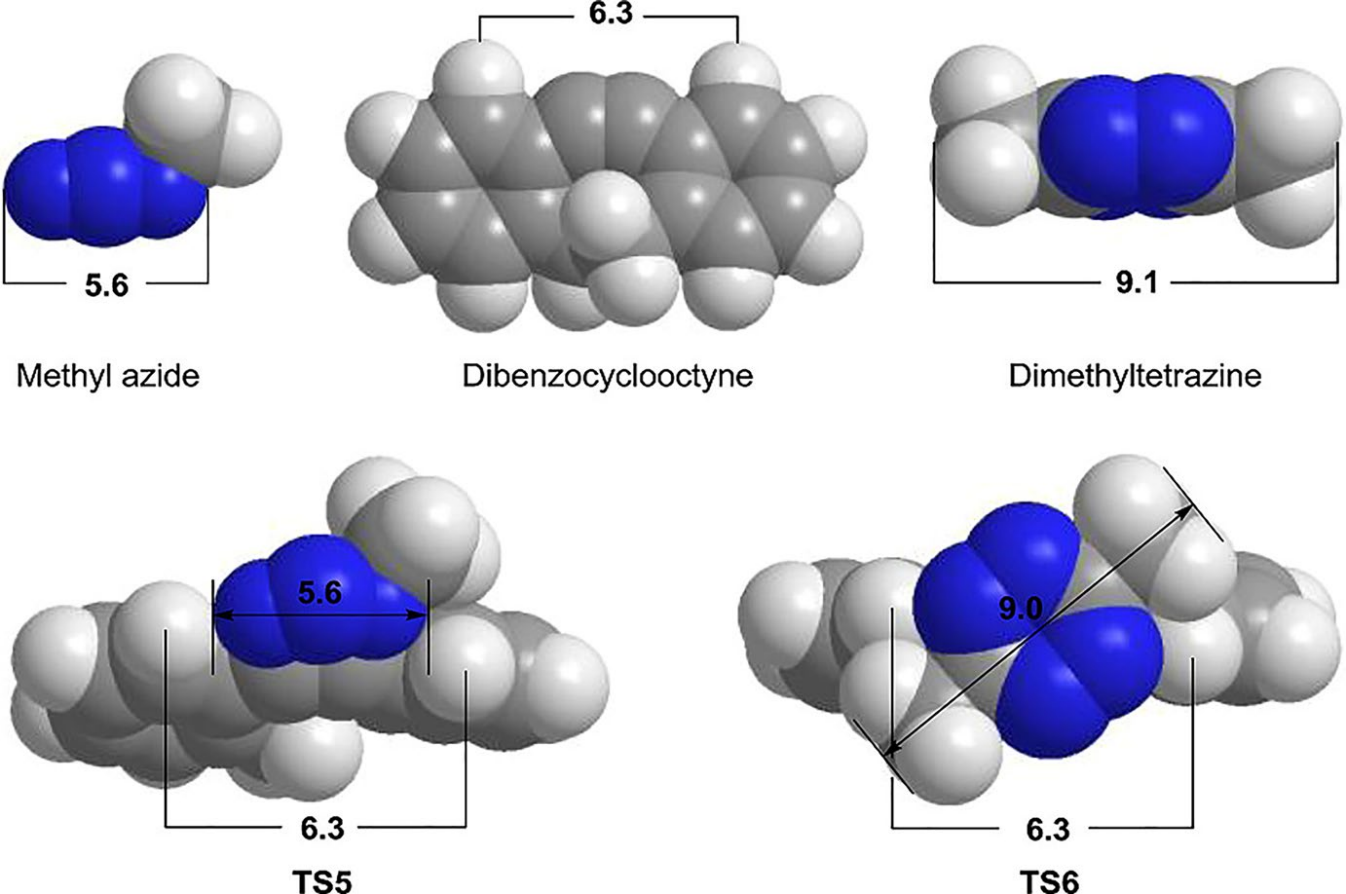
Space-filling models of dibenzocyclooctyne, methyl azide, dimethyltetrazine, and transition states **TS5** and **TS6** (distances in Å) [141]

1,2,4,5-Tetrazines rapidly react with sterically non-demanding cyclooctynes [138], particularly **BCN** [139, 140]. Houk and coworkers demonstrated that the steric demand of the flagpole hydrogens in dibenzocyclooctynes limits the reaction between 1,2,4,5-tetrazines and these dienophiles (Fig. 53) [141].

**Fig. 54:**
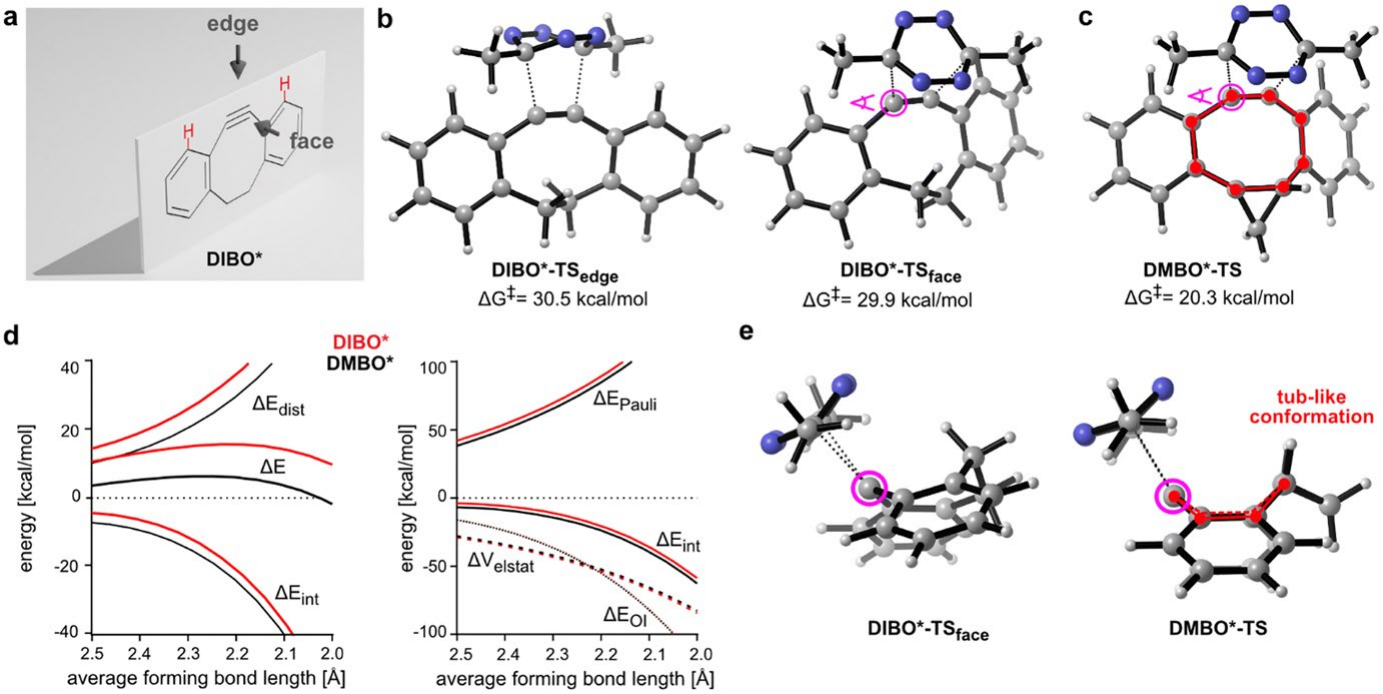
DFT calculations for TSs of DIBO* and DMBO* in an iEDDAC reaction with **MeMe-Tet**. **a** Schematic representation of the face and edge approach for dibenzoannulated cyclooctynes. **b** Transition state geometries and Gibbs free energy barriers for the face and edge approach of **MeMe-Tet **on DIBO*, calculated using M06-2X-D3/6–311 + G(d,p) SMD(water). Forming bond lengths are given in Å. **c** Transition state geometry and Gibbs free energy barrier for the reaction of **MeMe-Tet** and DMBO* calculated using M06-2X-D3/6–311 + G(d,p) SMD(water). Forming bond lengths are given in Å. **d** DIA (left side) and EDA (right side) along the intrinsic reaction coordinate for the reaction of **MeMe-Tet** with DIBO* or DMBO*. The analysis was performed in ADF using M06-2X-D3/TZ2P on M06-2X-D3/6–311 + G(d,p) SMD(water) calculated geometries. **e** View along the dibenzocyclooctyne triple bond for the face transition state of DIBO* with **MeMe-Tet** and DMBO* with **MeMe-Tet,** as indicated in purple in (**b**) and (**c**). Tub-like conformation of the DMBO*-TS is highlighted [71]. *iEDDAC* Inverse electron-demand Diels-Alder cycloadditions

However, in 2019, Lang and coworkers showed that fusing a cyclopropane ring to the backbone of dibenzocyclooctynes significantly increases their reactivity toward tetrazines [142]. The underlying mechanism of this increased reactivity was recently elucidated [71]. The integration of the cycloalkane enables the dienophile to adopt a tub-like conformation, resulting in reduced steric hindrance (Fig. 54).

### Triazines

1,2,4-Triazines have emerged as potential substitutes for 1,2,4,5-tetrazines [143]. Though these dienes show less reactivity, their stability is enhanced. The reduced reactivity is largely credited to the elevated LUMO energy of this particular azine [143, 144].

**Fig. 55:**
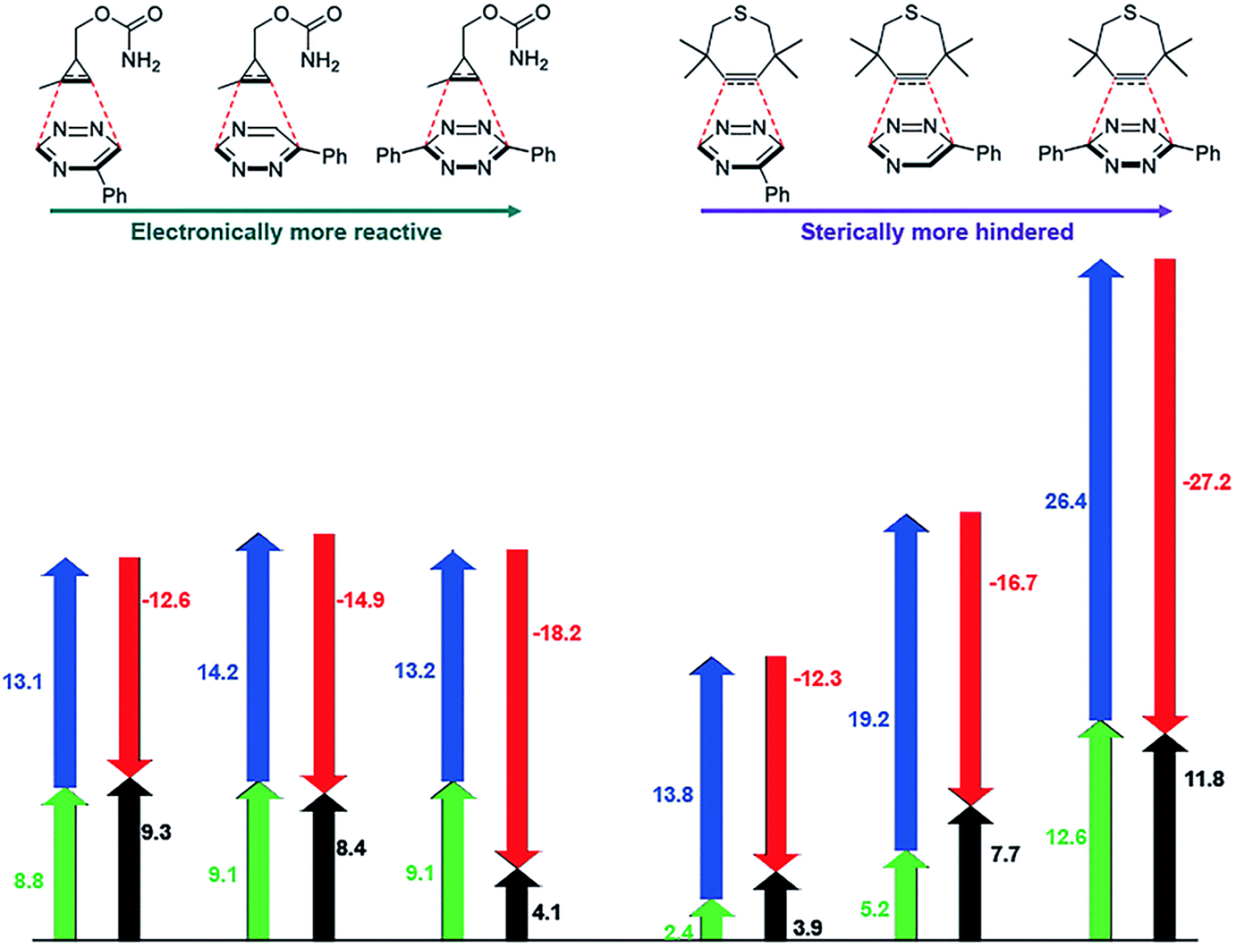
Distortion/interaction analysis of factors controlling mutually orthogonal cycloadditions. Black arrows are activation potential energies, green and blue arrows are distortion energies of dienophile and diene, respectively, and red arrows are interactions energies. All values are given in kcal mol^−1^. Reprinted with permission from [145]

Building on this understanding, Prescher and coworkers realized that the isomeric 1,2,4-triazines showcase distinct reactivities when paired with appropriate dienophiles [145]. Specifically, they observed that the 5-phenyl-1,2,4-triazine exhibits rapid reaction rate with TMTH, a 7-membered ring alkyne known for its exceptional reactivity but steric hindrance [146]. Conversely, the 6-substituted 1,2,4-triazine and the disubstituted 1,2,4,5-tetrazines showed no reactivity with this alkyne. Through DIA, it was revealed that the lack of reactivity in the latter was due to increased distortion energies, a consequence of steric hindrance (Fig. 55). Exploiting these variances in reactivity, the authors successfully devised orthogonal pairs of bioorthogonal reactions.

**Fig. 56:**
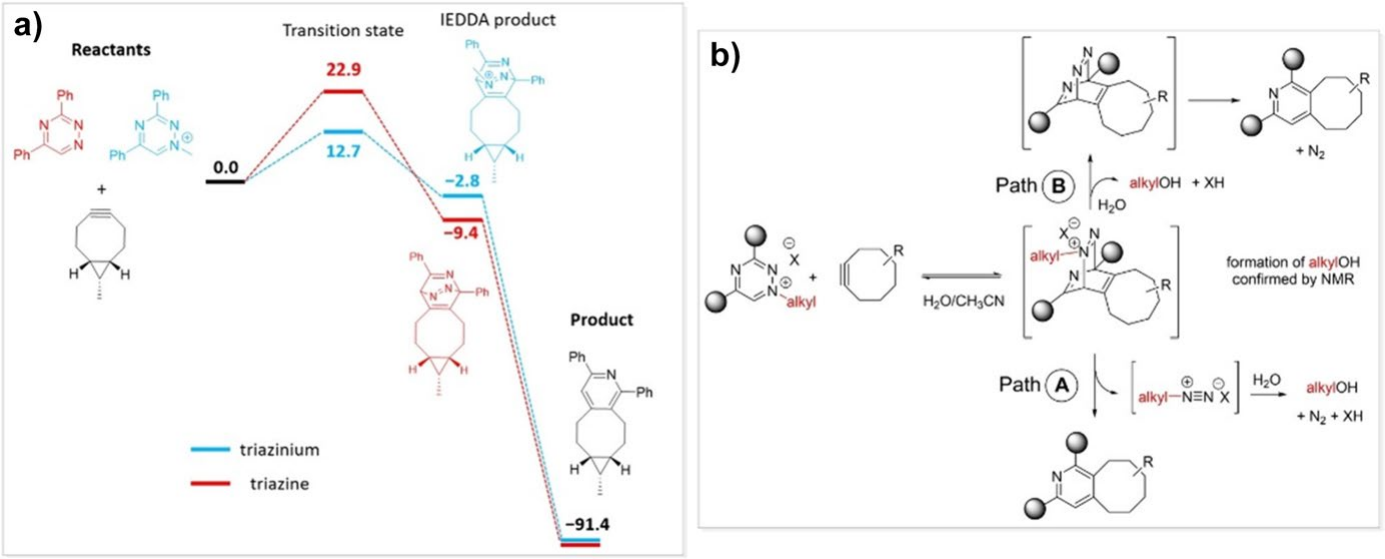
**a** The calculated (B3LYP/6-31 + G(2d,p)/CPCM) relative free energies (kcal mol^−1^) of the reactants, transition-state structures, and products of the IEDDA reaction of **Trz1** and **Trz**^**+**^**1** with **endo-BCN** (the hydroxymethyl group was substituted with a methyl group for simplification). **b** Proposed reaction mechanism of the triazinium ligation.* NMR* Neutron magnetic resonance Reprinted with permission from [147]

Vrabel and his team introduced N1-alkylated 1,2,4-triazinium salts as potential bioorthogonal reactants [147]. Specifically, the 3,5-disubstituted 1,2,4-triazinium derivatives demonstrated rapid reactivity with cyclooctynes such as **BCN**, while maintaining good stability. Remarkably, the reactivity of this highly electron-deficient diene surpasses the triazine compound by three orders of magnitude. Intrigued by this, the reaction mechanism was explored computationally (as depicted in Fig. 56). The primary question posed by the researchers pertained to the retro-Diels–Alder reaction that follows the initial cycloaddition. Two plausible pathways were considered: firstly, where the N-alkyl group undergoes hydrolysis, followed by the retro-Diels–Alder reaction involving N_2_; and secondly, where the alkylated N_2_ is eliminated, succeeded by dissociation. However, the computational analyses failed to offer definitive conclusions.

### Ortho Quinones

**Fig. 57:**
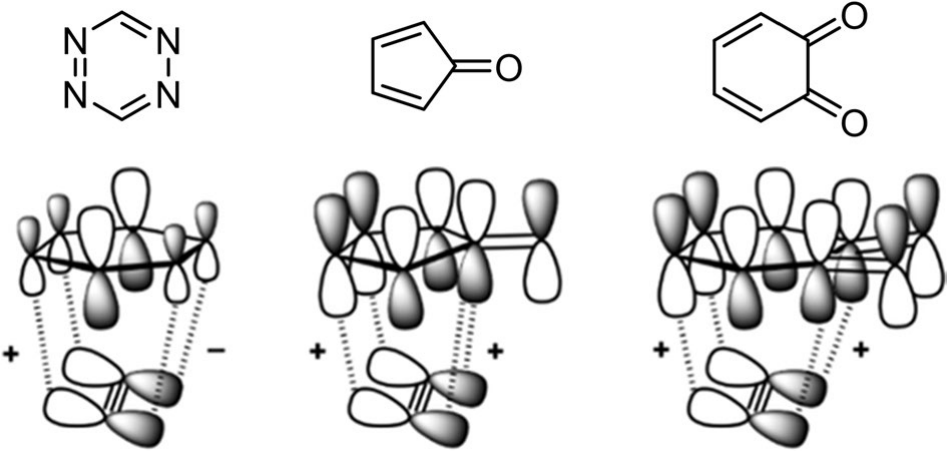
Schematic illustration of the constructive (+) and destructive (−) secondary orbital interactions between the HOMO_-1_ of **BCN** and the LUMOs of **tetrazine**, **cyclopentadienone**, and **1,2-quinones**. Adapted with permission from [113], licensed under CC-BY 4.0

Ortho-quinones have been recognized as dienes for bioorthogonal click reactions when paired with suitable dienophiles. **BCN** was employed by van Delft and his team in a strain-promoted oxidation-controlled cyclooctyne 1,2-quinone cycloaddition (SPOCQ) [148]. Zuilhof and associates, using both experimental and computational methods, explored the kinetics of reactions between various cyclooctynes and 1,2-quinones [149]. Notably, while 1,2,4,5-tetrazines favor reactions with *trans*-cyclooctenes over cyclooctynes, 1,2-quinones exhibit a contrasting preference. Levandowski et al. undertook a computational investigation to shed light on this peculiar reactivity [150]. Employing DIA and EDA, they demonstrated increased orbital interaction when 1,2-quinones reacted with alkynes, as opposed to alkenes. However, this trend was not observed with tetrazines or cyclopentadienones. The phenomenon was attributed to a secondary orbital interaction. The out-of-plane π-orbital of the alkyne, which is not directly involved in bond formation, interacts beneficially with the LUMO of 1,2-quinones. Such an interaction is absent when pairing with tetrazines or cyclopentadienones (Fig. 57).

Zuilhof and coworkers presented the reaction occurring between cyclopropenes and 1,2-quinones [148]. A computational examination indicated a preference for the endo transition state. The DIA further clarified that this preference arises from a reduced distortion energy.

### Cyclopentadiene Derivatives

**Fig. 58:**
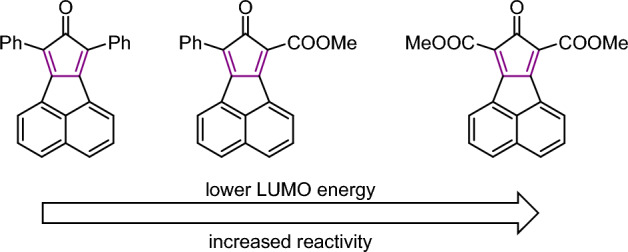
Cyclopentadienones investigated by Wang and coworkers [152]. The reactive diene is highlighted in purple

Several dienes based on cyclopentadiene have been suggested for use in bioorthogonal reactions. Cyclopentadienones react quickly with cyclooctynes like **BCN**. Following the initial cycloaddition, CO elimination results in the formation of benzene, allowing this process to act as a prodrug for CO [151]. Wang and colleagues introduced a strained cyclopentadienone as a probe that becomes fluorescent upon activation [152]. Through computational studies, they demonstrated that electron-withdrawing substituents accelerate these reactions, consistent with the expectations of FMO theory in inverse electron demand Diels–Alder reactions (Fig. 58).

**Fig. 59:**
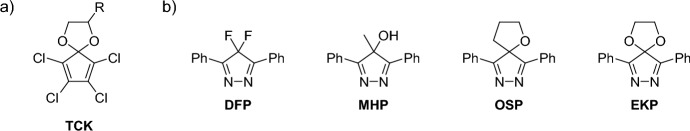
**a** Tetrachlorocyclopentadiene ketals. **b** Bioorthogonal *4H*-pyrazoles

Houk, Murphy, and coworkers proposed tetrachlorocyclopentadiene ketals (TCK) as bioorthogonal dienes (Fig. 59a) [153]. Computational studies indicated that they react rapidly with *trans*-cyclooctenes and less sterically demanding cyclooctynes like **BCN**. However, their reactivity with cyclooctynes such as **DIBO** is limited due to steric hindrance (Fig. 59). Subsequent experimental research corroborated these findings.

Levandowski et al*.* showcased that *4H*-pyrazoles have the potential to function as bioorthogonal reactants, undergoing reactions with strained alkynes like **BCN** [154]. The initial reactants of this class incorporated difluoro substitutions to accelerate the reaction, as exemplified by DFP (Fig. 59b). However, a drawback to this approach was the decreased stability of these reactants in biological environments.

To counter this limitation, Raines and his team suggested alterations aiming at enhancing stability without compromising the high reactivity [155]. They introduced three new reactants, namely **MHP**, **OSP**, and **EKP** (Fig. 59b). By replacing the fluoro groups with alkyl or oxy groups, the destabilizing hyperconjugative antiaromaticity of the molecule is reduced [156], thereby enhancing its stability. Incorporating spiro compounds is another strategy they employed to diminish steric interference with the approaching dienophile, which in turn accelerates the reaction. Among the trio, **OSP** emerged as the most reactive diene.

It was observed that symmetrically substituting a *4H*-pyrazole leads the molecule to adopt a slight envelope-like distortion at its saturated center. For **MHP** and **OSP**, their energy-minimized structures revealed the saturated centers to be puckered by 2.6° and 4.0°, respectively, from the plane of the diene π-system. This particular distortion, especially visible in OSP, increases its reactivity. This is because the puckering brings the molecule closer to the geometric requirements of the syn Diels–Alder transition state, reducing the necessary conformational distortion for the reaction to proceed [156].

## Cheminformatics and Machine Learning Approaches

Given the increasing prominence of machine learning and cheminformatics in advancing the field of chemistry, the discussion on their integration in designing chemical tools certainly warrants its own section.

One method for designing new or enhanced chemical tools involves utilizing datasets to generate statistical or machine learning models capable of predicting reactivity or selectivity at significantly lower computational costs. In an ideal scenario, large datasets of experimentally measured data would be used for such purposes. However, in the area of bioorthogonal chemistry, comprehensive and systematic reactivity datasets are lacking. One primary reason is the complexity involved in preparing numerous bioorthogonal reactants, which limits the availability of derivatives. Reactivity data are also often collected in varied solvent systems, partly due to solubility challenges, and this variability is especially prominent in tetrazine reactions, which exhibit significant solvent effects. Varying temperatures further add to the complexity of these datasets.

In 2020, Ravasco and Coelho developed a predictive multivariate model specifically aimed at bioorthogonal inverse-electron demand Diels–Alder reactions [157]. They compiled a dataset from the literature, consisting of approximately 500 data points that encompassed 151 dienophiles and 108 dienes. These compounds were studied across various solvents and temperature conditions. Notably, the dienes included 1,2,4,5-tetrazines and 1,2,4-triazines, and the span of reactivity covered 12 orders of magnitude.

**Fig. 60:**
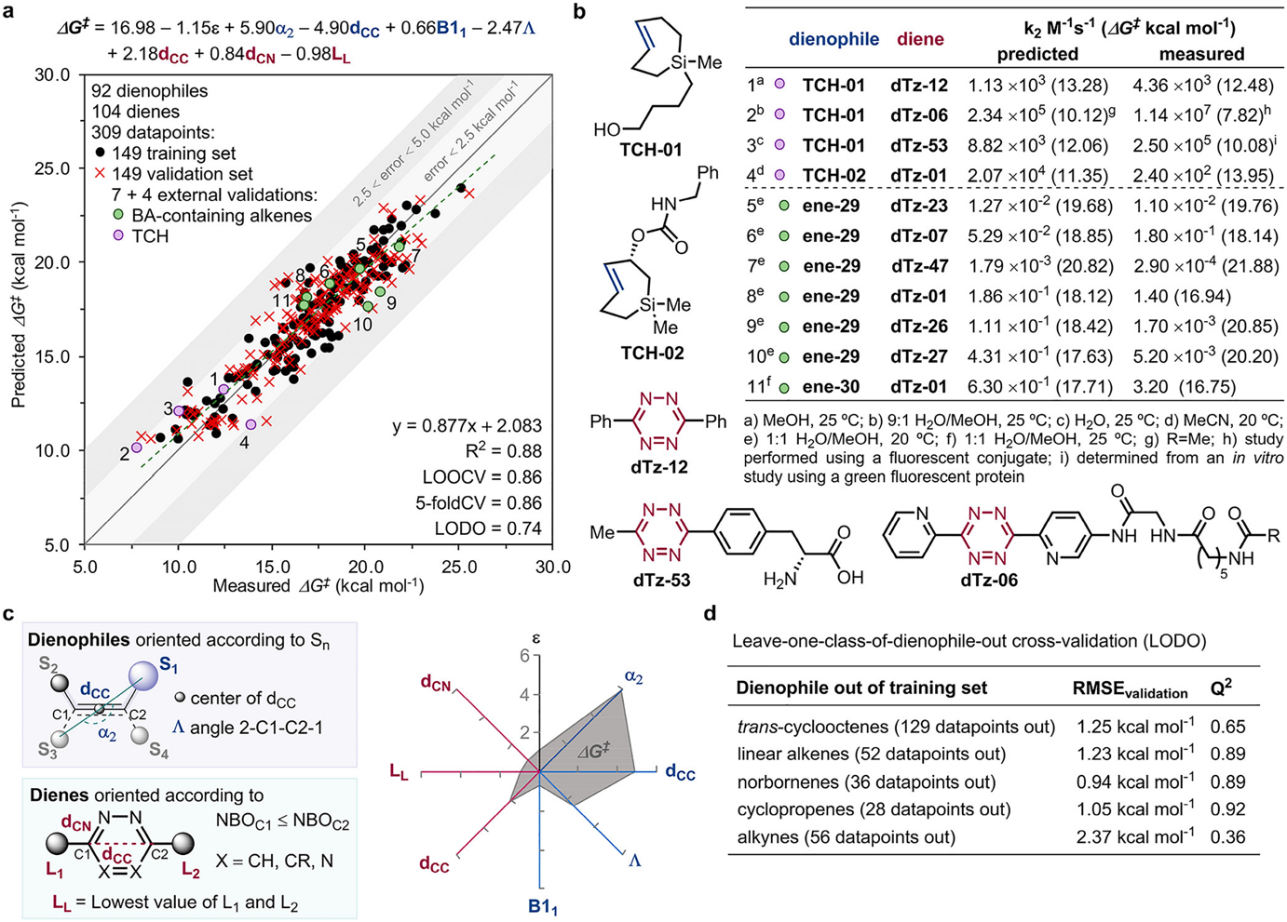
**a** Multivariate model for IEDDA reaction. **b** Predicted and measured second order rate constants for out-of-sample structures. **c** Parameters and visual analysis of the model terms contribution to **ΔG**^**‡**^ (each axis represents the absolute value of normalized coefficients of the corresponding parameter). **d** RMSE and Q^2^ values of LODO analysis.* LODO* Leave-one-dataset-one, *RMSE* root-mean-square error. Reprinted with permission from [157]. Copyright 2020 American Chemical Society

The model operates by calculating various descriptors at the DFT level using the M06-2X/6-31G(d) level for both reactants. These descriptors are then incorporated into the multivariate model to predict the free energy of activation (Fig. 60). A number of descriptors were identified as significant by the model. As expected, the dielectric constant of the solvent showed an inverse correlation with reactivity, aligning with the general understanding that more polar solvents accelerate Diels–Alder reactions. For the dienophile, several geometric and steric parameters were critical, along with the HOMO energy and NBO charges—findings that are intuitive for an inverse-electron-demand Diels–Alder reaction. Interestingly, for the diene, the model highlighted the importance of various geometric descriptors related to the ring structure, while orbital energies were not found to be significant. Leveraging this model, the researchers were able to predict reaction barriers with an accuracy within a few kcal/mol, using relatively inexpensive DFT calculations of the reactants.

In 2023, Stuyver and Coley employed a segment of a computational dataset comprising 10 million (3 + 2) cycloaddition barriers and energies of formation, previously prepared by the same research group [158], to develop a machine learning model for screening potential candidates for bioorthogonal click reactions [159]. Utilizing a graph neural network, they successfully predicted free energies of activation and formation within an accuracy range of 2–3 kcal/mol, using only the SMILES (simplified molecular-input line-entry system) input of the reactants and product. However, high reactivity alone is not sufficient for bioorthogonal applications. Reactants must also exhibit selective interactions with each other, and the reaction equilibrium should be irreversible. To assess bioorthogonal potential, Stuyver and Coley applied three criteria: (1) the activation barrier should be below 25 kcal/mol to enable rapid reactions; (2) the reverse reaction should possess an activation barrier above 28 kcal/mol to ensure that the equilibrium favors the product; and (3) the selected 1,3-dipole's reaction with a native (biological) dipolarophile should be sufficiently slower (higher activation barrier) to minimize background reactions in biological media. Using these criteria, they identified several promising candidates for further investigation [159].

Another promising strategy in computational bioorthogonal chemistry is the use of machine learning models to bridge the gap between low-level semiempirical calculations and high-level DFT predictions. In this approach, quick and computationally inexpensive semiempirical calculations are initially performed to obtain features like orbital energies or activation energies. These features are then fed into a machine learning model trained to predict high-level DFT activation energies. Grayson and coworkers recently demonstrated the effectiveness of this method in a study focused on Diels–Alder reactions [160]. While their research did include calculations for tetrazine/alkene cycloadditions, the study was primarily aimed at validating the technology itself rather than its applicability to bioorthogonal chemistry. Additionally, they did not explore derivatives commonly used in bioorthogonal reactions. Although this method has not been explicitly applied to bioorthogonal reactions yet, it holds considerable potential for future work in this field of computational chemistry.

## Conclusion

Computational organic chemistry has been instrumental in shaping the trajectory of bioorthogonal chemistry. In the area of (3 + 2) cycloadditions, Raines and Alabugin's groups have been at the forefront, pioneering the development and understanding of cyclooctyne/1,3-dipolar cycloadditions. Leveraging natural bond orbitals analysis and hyperconjugation as strategic tools, they refined this bioorthogonal reaction to great effect. The groups of Bickelhaupt and Houk have explored the intrinsic reactivity of cyclooctynes. Additionally, numerous researchers have utilized computational tools to elucidate and further improve these cycloadditions.

In the domain of [4 + 2] cycloadditions, tetrazine cycloadditions have been predominant. Here, computational tools have shed light on the intrinsic reactivity of this fascinating diene. Techniques like distortion/interaction analysis and energy decomposition analysis have paved the way for a better grasp of substituent effects and have been pivotal in crafting more effective click reactions. Beyond this, computational studies have also been crucial in optimizing [4 + 2] cycloadditions involving other dienes, such as 1,2-quinones and 1,2,4-triazines, and have led to the identification of multiple orthogonal bioorthogonal reactions.

Emerging trends in cheminformatics and machine learning hold immense promise. They offer exciting avenues that could potentially fast-track the evolution of these reactions.

In summary, the impact of computational organic chemistry on bioorthogonal chemistry has been profound and undeniable.

## Data Availability

This article is a review and does not involve the generation or analysis of any new data. For additional information regarding the data discussed, readers should contact the authors of the referenced works.
